# Expression of a unique *M. tuberculosis* DNA MTase Rv1509 in *M. smegmatis* alters the gene expression pattern and enhances virulence

**DOI:** 10.3389/fmicb.2024.1344857

**Published:** 2024-05-13

**Authors:** P. Manjunath, Javeed Ahmad, Jasmine Samal, Anshu Rani, Javaid Ahmad Sheikh, Sheeba Zarin, Yashika Ahuja, Anwar Alam, Seyed E. Hasnain, Nasreen Z. Ehtesham

**Affiliations:** ^1^Inflammation Biology and Cell Signaling Laboratory, National Institute of Pathology, New Delhi, India; ^2^Department of Biotechnology, Jamia Hamdard, New Delhi, India; ^3^Molecular Biology Section, Laboratory of Immune System Biology, National Institute of Allergy and Infectious Diseases, National Institutes of Health, Bethesda, MD, United States; ^4^Kusuma School of Biological Sciences, Indian Institute of Technology, New Delhi, India; ^5^Department of Life Science, Sharda School of Basic Sciences and Research, Sharda University, Greater Noida, Uttar Pradesh, India; ^6^Department of Biotechnology, Sharda School of Engineering Sciences and Technology, Sharda University, Greater Noida, Uttar Pradesh, India; ^7^Department of Biochemical Engineering and Biotechnology, Indian Institute of Technology, New Delhi, India

**Keywords:** BCG, macrophage activation, phagolysosomal escape, RNA-seq, signature sequence, transcription regulator, T-regulatory cells

## Abstract

*Mycobacterium tuberculosis* (*M. tb*) genome encompasses 4,173 genes, about a quarter of which remain uncharacterized and hypothetical. Considering the current limitations associated with the diagnosis and treatment of tuberculosis, it is imperative to comprehend the pathomechanism of the disease and host-pathogen interactions to identify new drug targets for intervention strategies. Using *in-silico* comparative genome analysis, we identified one of the *M. tb* genes, Rv1509, as a signature protein exclusively present in *M. tb*. To explore the role of Rv1509, a likely methyl transferase, we constructed a knock-in *Mycobacterium smegmatis* (*M. smegmatis*) constitutively expressing Rv1509 (Ms_Rv1509). The Ms_Rv1509 led to differential expression of many transcriptional regulator genes as assessed by RNA-seq analysis. Further, *in-vitro* and *in-vivo* studies demonstrated an enhanced survival of Ms_Rv1509 inside the host macrophages. Ms_Rv1509 also promoted phagolysosomal escape inside macrophages to boost bacterial replication and dissemination. *In-vivo* infection studies revealed that Ms_Rv1509 survives better than BCG and causes pathological manifestations in the pancreas after intraperitoneal infection. Long-time survival of Ms_Rv1509 resulted in lymphocyte migration, increased T regulatory cells, giant cell formation, and likely granuloma formation in the pancreas, pointing toward the role of Rv1509 in *M. tb* pathogenesis.

## Introduction

Tuberculosis (TB) is one of the deadliest infectious diseases in the world and infects about one-quarter of the world's population (WHO, 2021). Despite the majority of the population being asymptomatic, 10 million people suffered from active disease, and 1.3 million lost their lives to TB in 2020 (Chakaya et al., 2021). The COVID-19 pandemic-mediated TB service disruptions resulted in a substantial fall in TB case notifications with a surge in death rates demanding a refocus on TB control strategies with an emphasis on novel diagnostics and vaccines (Jeremiah et al., 2022; Shariq et al., 2022b). The efficacy of a currently available vaccine, Bacillus Calmette-Guerin (BCG), is variable against pulmonary TB in adults, though studies to improve BCG efficacy and immune response are underway (Schaible et al., 2017; Darrah et al., 2020; Sheikh et al., 2020). A better understanding of host immune evasion by *Mycobacterium tuberculosis* (*M. tb*) and elucidation of pathways involved in the survival of the pathogen could provide important insights in designing strategies for better diagnosis and treatment of TB (Sharma N. et al., 2021; Nehvi et al., 2022; Shariq et al., 2022a).

*M. tb* genome codes for a total of 4,173 genes encoding 4,136 proteins (Cole et al., 1998; Kapopoulou et al., 2011). Approximately 25% of these are annotated as hypothetical proteins whose functions need to be ascertained to understand the functionality of *M. tb* and its pathogenesis. Relentless efforts are underway to unravel the function(s) of uncharacterized proteins of *M. tb* to gain insights into its immunoregulatory role and pathogenesis (Ahmad et al., 2018, 2020; Arora et al., 2020a; Sharma et al., 2020, 2022; Ali et al., 2021; Ehtram et al., 2021; Shariq et al., 2021; Sharma N. et al., 2021; Sharma T. et al., 2021). The astounding aspect of reductive evolution in mycobacteria, along with gain in pathogenesis, suggests complex levels of gene regulation and coordination in pathogens like *M. tb* (Ahmed et al., 2008; Rahman et al., 2014). Therefore, unraveling these multilayered gene networks to elucidate such genes that are exclusively present in pathogenic *M. tb* and absent in non-pathogenic strains is crucial to understanding *M. tb* pathogenicity (Kohli et al., 2012; Singh et al., 2014).

It is becoming increasingly evident that epigenetic modifications play an important role in modulating *M. tb* virulence, survival, and pathogenesis (Temmerman et al., 2004; Chiner-Oms et al., 2019; DiNardo et al., 2020). DNA methyltransferases (MTases) are important key players in regulating the expression of genes at the DNA level, impacting gene expression patterns both at the bacterial and host levels (Adhikari and Curtis, 2016). We previously showed the presence of 121 MTases in the *M. tb* H_37_Rv genome, of which many represent hypothetical and uncharacterized MTases (Grover et al., 2016). Intriguingly, approximately 70% of these MTases, which represent >3% of the total proteome, are S-Adenosyl-l-methionine (AdoMet)-dependent methyltransferases (MTases) (AdoMetS). The presence of such a high number of MTases in the *M. tb* genome, compared to other mycobacterial species, implicates a diverse epigenetic role in regulating the virulence and pathogenicity of *M. tb*. Our previous findings have demonstrated that there are specific methyltransferases such as Rv1509, which are present only in the *M. tb* complex and the recently sequenced *Mycobacterium riyadhense*, and not in other pathogenic, non-pathogenic, and opportunistic mycobacterial species, emphasizing the importance of methyltransferases in pathogenicity (Grover et al., 2016). Taking clues from these findings, we proposed that the Rv1509 gene, being a pathogen-specific methyltransferase, is expected to modulate the gene regulatory networks to influence *M*. tb virulence and pathogenicity. *M.smegmatis* mc^2^155 shares 2547 orthologs genes with *M. tb* H_37_Rv (Jiang et al., 2022) and serves as a surrogate model to study the pathophysiology of *M. tb* genes (Anes et al., 2003; Sweeney et al., 2011; Lelovic et al., 2020). In this study, we describe the morphological, physiological, and immunological attributes of this gene using a knock-in *M. smegmatis* constitutively expressing the *M. tb* Rv1509 gene. We further explore the possible role of Rv1509 in host-pathogen interactions using *in-vitro* (cell lines) and *in-vivo* (mice) studies.

## Results

### Expression of *Mycobacterium tuberculosis* Rv1509 protein retards growth, modifies cell wall, inhibits septum formation, and alters proteins expression pattern in *M. smegmatis*

Computational analysis of *Mycobacterium tuberculosis* Rv1509 protein sequence revealed the presence of an S-adenosylmethionine binding motif, methyltransferase motif, and DNA binding leucine zipper motif (Supplementary Figure S1A). The presence of these motifs pointed to the likelihood of it being a DNA methyltransferase. Further, to characterize the methyltransferase activity of *M. tb* Rv1509 protein, we expressed and purified recombinant Rv1509 protein in *Escherichia coli* and purified via metal-affinity chromatography to high purity (Supplementary Figure S1B). Methyltransferase activity was assessed using a colorimetric-based ELISA kit (EPIGENTEK# P-3139-48) with gradient concentrations of Rv1509 protein (1, 2, 4, and 8 μg/ML). BSA was used as a negative control. Our results confirmed the *in-silico* prediction and demonstrated that the Rv1509 protein has SAM-dependent DNA methyltransferase activity (Supplementary Figure S1C). Since Rv1509 displayed a DNA binding motif, a DNA binding assay using fluorescence emission spectra was performed. As the DNA concentrations increased, the intensity of protein absorbance decreased, which indicated that this protein binds non-specifically to DNA (Supplementary Figures S1D, E). A deep learning-based AlphaFold (PMID#) structural prediction model of Rv1509 protein showed that the protein is highly structured with alpha helix and beta sheet folds. The pLDDT scores generated by AlphaFold described a model of very high quality (pLDDT > 90) (Supplementary Figure S1F).

**Figure 1:**
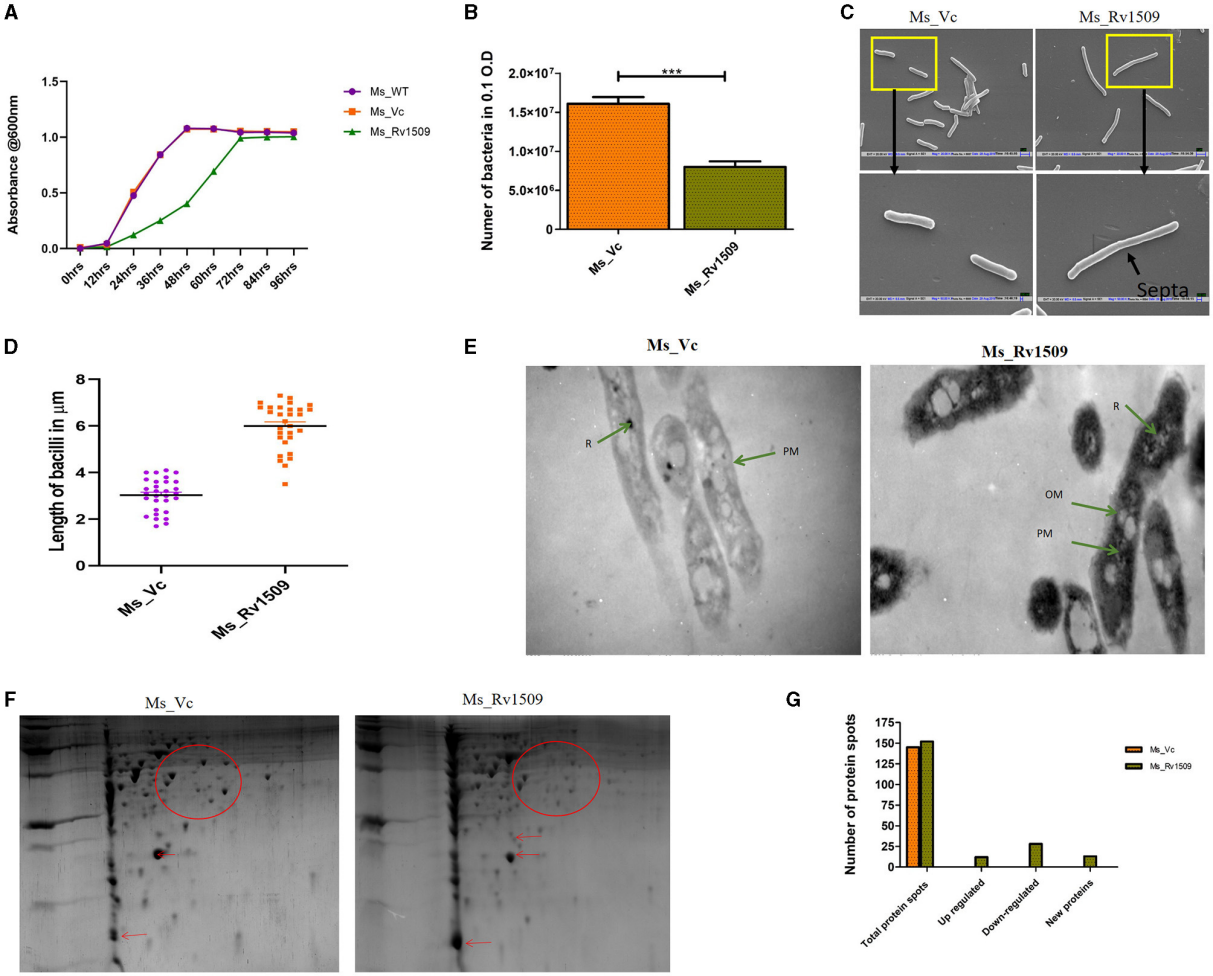
Ectopic expression of Rv1509 alters bacterial length and retards growth of *Mycobacterium smegmatis*. **(A)** Comparison of growth kinetics between Ms_WT, Ms_Vc, and Ms_Rv1509 grown in Middlebrook 7H9 medium supplemented with 0.05% Tween 80, 0.5% glycerol, and 10% OADC. **(B)** A bar graph showing the total number of bacilli present in 0.1 OD of Ms_Vc and Ms_Rv1509 cultures. **(C, D)** SEM analysis confirmed the increased length of Ms_Rv1509 compared to Ms_Vc (Upper panel 20 K and Lower panel 50 K magnification). **(E)** TEM analysis revealed the outer cell membrane (OM) modification and increased ribosomal content (R) in Ms_Rv1509 (Direct magnification 10,000X). **(F)** A 2D gel electrophoresis analysis showing differential expression of proteins between Ms_Vc and Ms_Rv1509. **(G)** Bar graph showing the upregulated proteins, downregulated proteins, and new protein spots of Ms_Rv1509 in comparison with Ms_Vc by PD Quest software analysis. (*p* ≤ 0.001= ***).

Since *M. smegmatis* serves as a good surrogate model to study the pathophysiology of *M. tb* genes (Lelovic et al., 2020), we produced a knock-in of the Rv1509 gene, confirmed with colony PCR, and verified the expression of the protein in *M. smegmatis* by Western blot. Growth curve analysis demonstrated that the doubling time of Ms_Rv1509 increased from 4 h to 12 h as compared to vector control (only pST-Ki electroporated in *M. smegmatis*) and wild-type *M. smegmatis* (Figures 1A, B), an attribute of the slow-growing pathogenic mycobacteria. In addition, the colony-forming unit per ml (CFU/ml) assay revealed that 0.1 OD (600 nm) had a lesser number of recombinant *M. smegmatis* compared to the vector control bacilli (Figure 1B).

The classical rod shape of *M. tb* ranges between 1–5 μm in length (Vijay et al., 2017). To further probe into the increased length of Ms_Rv1509, a scanning electron microscopy (SEM) analysis was performed. The SEM analysis revealed an incomplete septa formation in Ms_Rv1509 compared to the control, implying an increase in the overall length of the bacilli, possibly due to a defect in septa formation (Figures 1C, D). The length of the Ms_Rv1509 bacilli ranged between 3.5 μm to 9.0 μm whereas the length of the Ms_Vc varied between 2.0 μm to 5.0 μm (Supplementary Figures S2A, B). As we observed changes in the growth kinetics and bacterial morphology, we further investigated to check whether there was any change in the internal membrane structure of recombinant bacilli. When comparing Ms_Rv1509 to Ms_Vc, transmission electron microscopy (TEM) revealed considerable differences in the cell wall and cytoplasm (Figure 1E). The thickness of the cell wall of Ms_Rv1509 was notably higher than the control bacilli. An intense electron-dense layer, which usually refers to ribosomal content (Yamada et al., 2015), was higher in Ms_Rv1509 than the Ms_Vc, suggesting a probable upregulation of translational machinery of recombinant *M. smegmatis* expressing Rv1509 protein. Protein expression maps of *M. smegmatis* expressing Rv1509 and *M. smegmatis* having empty vectors were obtained by 2D gel electrophoresis (Figures 1F, G). There was a significant difference in the protein expression profile in Ms_Rv1509 compared to that of Ms_Vc. Comparing the protein maps on 2D gels by PD QUEST software revealed a differential expression of proteins in the Ms_Rv1509. A cursory analysis revealed a total of 145 spots in Ms_Vc compared to 152 spots in Ms_Rv1509. Differential expression analysis reveals that there were 12 upregulated proteins in Ms_Rv1509, along with 28 downregulated proteins. Some new proteins were found to be expressed by Ms_Rv1509 (*n* = 13), while some proteins were absent in Ms_Rv1509 (*n* = 13).

### *M. smegmatis* expressing Rv1509 protein upregulates transcription factors, transcriptional regulators, metabolic process, and translational genes

After observing significant changes in the growth kinetics and morphology of *M. smegmatis* in the presence of Rv1509 protein, we were curious to compare the protein expression patterns in Rv1509 protein expressing *M. smegmatis* to that of control.

**Figure 2:**
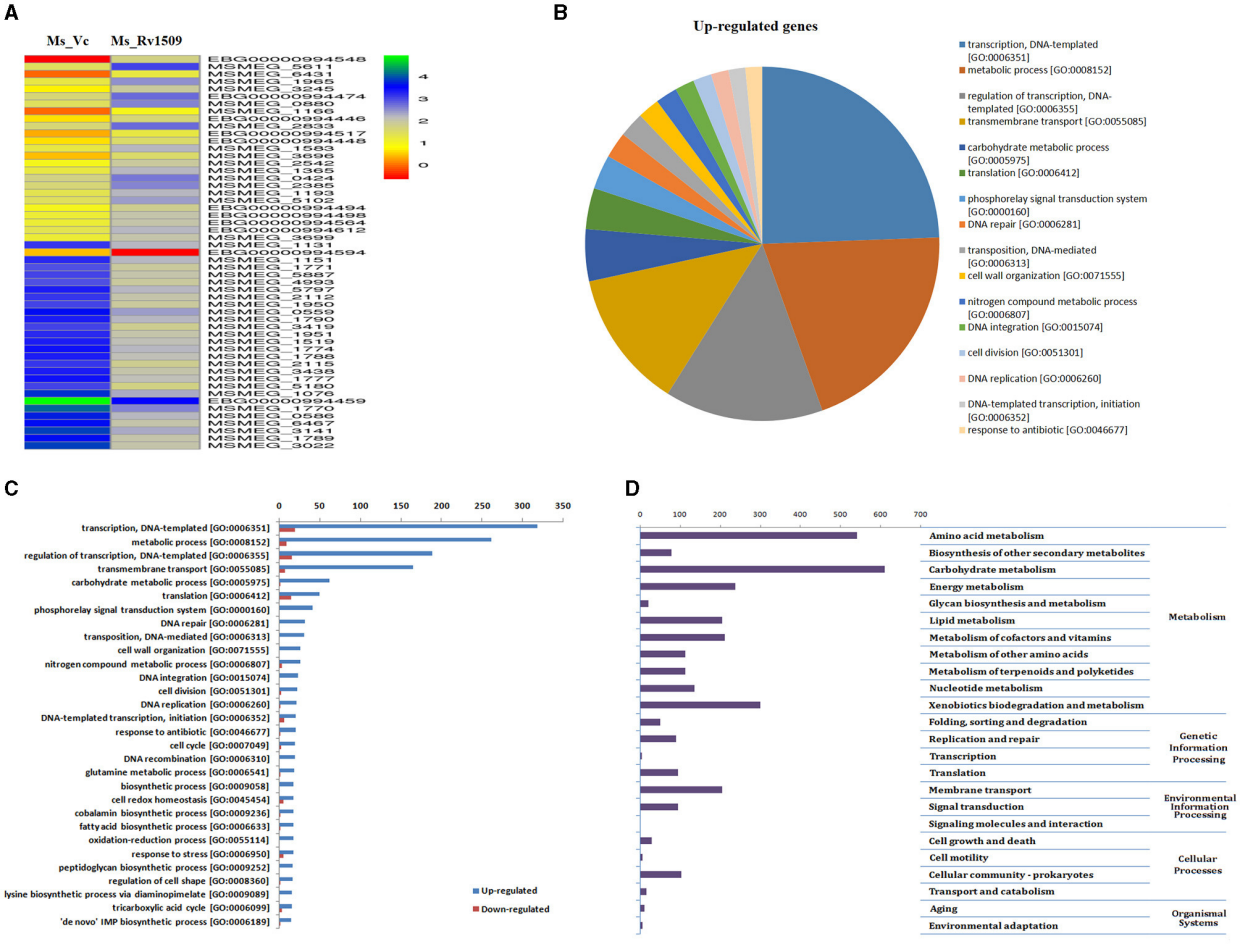
RNA-seq analysis depicting differential expression of genes in Ms_Vc and Ms_Rv1509. **(A)** The Heat map analysis of Ms_Rv1509 and Ms_Vc shows the top 25 up and downregulated genes. **(B)** Pie charts showing the upregulated genes in Ms_Rv1509. **(C)** The Box plot shows the statistical analysis of differentially upregulated and downregulated genes between Ms_Vc and Ms_Rv1509. **(D)** KEGG pathway analysis shows the differentially regulated genes involved in different biological pathways, including amino acid metabolism, carbohydrate metabolism, lipid metabolism, xenobiotics biodegradation, and membrane transport.

Based on large-scale variation in protein expression, we further analyzed the differential gene expression pattern between Ms_Rv1509 and Ms_Vc by RNA-seq technology. The bacterial cultures were grown up to the mid-log phase (O.D_600_ = 0.4) under optimal growth conditions, followed by RNA isolation and RNA sequencing. It was intriguing to observe that the expression of signature protein Rv1509 in *M. smegmatis* led to the upregulation of more than 439 genes (cut off Log2). The heatmap representing the upregulation and downregulation of the genes in Ms_Vc and Ms_Rv1509 is shown in Figure 2A. The pie chart and gene ontology (GO) analysis revealed that genes involved in the transcriptional and translational machinery were upregulated in Ms_Rv1509 compared to Ms_Vc (Figures 2B, C). The transcriptional regulatory network plays a crucial role in controlling the mycobacterial virulence and pathogenesis inside the host (Table 1). Interestingly, a conserved hypothetical gene MSMEG_6431 in *M. smegmatis* (the gene is orthologous to the ESPR gene of *M. tb*) showed an approximately 4-fold increase in knock-in *M. smegmatis* compared to the control. Notably, RNA-seq data revealed multiple tRNAs (coding genes) (Table 2) and non-coding tRNAs (Table 3) being upregulated in Ms_Rv1509 compared to Ms_Vc, indicating a redirection of its own translational machinery to promote its survival inside the host. This further supports our TEM analysis, which showed an increased ribosomal content in Ms_Rv1509. KEGG pathway analysis of RNA-seq data suggested that many genes belonging to different metabolic pathways were upregulated in Ms_Rv1509. Genes involved in amino acid metabolism and carbohydrate metabolism were significantly upregulated in Ms_Rv1509 (Figure 2D). Very interestingly, more than 60% of the genes (>2,900 genes) involved in catalytic activity, which are important in molecular functions, were upregulated—the highest number in terms of upregulation (Supplementary Figure S3).

**Table 1:** A list of upregulated transcriptional regulators (genes) in Ms_1509 as compared to Ms_Vc by RNA-Seq analysis.

	**Ms_Vc**		**Ms_Rv1509**					
**Gene ID**	**Coverage**	**FPKM**	**Coverage**	**FPKM**	**Fold change**	**Log 2 fold change**	**Gene description**	**Presence in Mtb**
MSMEG_6431	0	0	0	18.10918	19.10918	4.256194	Conserved hypothetical protein	ESPR in Mtb
MSMEG_0735	51.11312	13.01782	278.7552	96.2711	6.939102	2.794749	Putative transcriptional regulator	Absent in Mtb
MSMEG_3840	11.07064	2.819542	66.50304	25.2523	6.873153	2.780972	LysR-family protein transcriptional regulator	Absent in Mtb
MSMEG_2794	26.87934	6.845803	136.7436	51.1257	6.643769	2.732002	Transcriptional regulator, GntR family protein	Rv0494
MSMEG_2538	39.00233	11.06621	215.8858	77.19282	6.480311	2.696063	MarR-family protein transcriptional regulator	Rv2887
MSMEG_0676	63.23464	16.10501	319.1155	102.4903	6.050291	2.597004	Putative transcriptional regulatory protein	Rv1776
MSMEG_0538	195.2155	50.48492	943.2541	304.3367	5.930605	2.568179	Regulatory protein, MarR	Absent in Mtb
MSMEG_2030	33.11953	8.575768	154.1044	51.79226	5.51311	2.462866	Transcriptional regulator, TetR family protein	Absent in Mtb
MSMEG_5651	30.23369	7.700111	103.8256	36.15379	4.270496	2.094404	Transcriptional regulator, LuxR family protein	Absent in Mtb
MSMEG_2386	24.66809	6.282629	93.83761	29.82288	4.232384	2.08147	Transcriptional regulator, IclR family protein	Rv2989
MSMEG_1953	277.0047	70.54936	1061.263	351.5761	4.927732	2.300924	Transcription factor WhiB	Rv3197A

**Table 2:** Top upregulated coding tRNAs and chaperonin in *Mycobacterium smegmatis* containing *Rv1509* gene by RNA-seq data analysis.

	**Ms_Vc**		**Ms_Rv1509**				
**Gene ID**	**Coverage**	**FPKM**	**Coverage**	**FPKM**	**Fold change**	**Log 2 fold change**	**Gene description**
MSMEG_1965	71.14286	18.11912	968.5065	307.8046	16.15161	4.013606	tRNA met
MSMEG_3245	18.44156	4.696815	283.9984	90.25861	16.01923	4.001733	tRNA leu
MSMEG_0880	105.762	27.4209	1,078.366	342.7195	12.0939	3.596207	Chaperonin GroL
MSMEG_1166	0	0	16.69481	10.24884	11.24884	3.491704	tRNA tyrosine
MSMEG_2833	192.28	48.97112	1,664.68	529.0581	10.60729	3.406984	tRNA Val
MSMEG_1583	66.25077	17.11976	496.5206	163.5158	9.079358	3.18259	Chaperonin GroL

**Table 3:** List of upregulated non-coding tRNA genes in Ms_1509 compared to Ms_Vc according to RNA-seq analysis.

		**Ms_Vc**		**Ms_Rv1509**			
**Gene ID**	**Gene name**	**Coverage**	**FPKM**	**Coverage**	**FPKM**	**Fold change**	**Log2 fold change**
EBG00000994548	tRNA	0	0.22847	0	52.31762	228.9912	7.839148334
EBG00000994474	tRNA	123.944	42.23972	1662.413	528.3375	12.50807	3.644787599
EBG00000994446	tRNA	16.38812	4.173833	142.1929	45.19086	10.82718	3.436586261
EBG00000994517	tRNA	0	2.071974	0	20.49808	9.893018	3.306410767
EBG00000994448	tRNA	16.58904	4.225004	0	39.34232	9.311784	3.219057533
EBG00000994494	tRNA	38.42329	9.785892	236.1464	75.05058	7.669263	2.939087859
EBG00000994498	tRNA	58.84507	14.98704	360.7606	114.6546	7.650251	2.935507174
EBG00000994564	tRNA	0	15.16994	362.4384	115.1879	7.593167	2.924701661
EBG00000994612	tRNA	83.20548	21.19131	503.9871	160.174	7.558474	2.918095064
EBG00000994462	tRNA	66.2973	16.88503	396.9341	126.1511	7.471181	2.901336395
EBG00000994455	tRNA	93.9589	23.93006	489.8904	155.6939	6.506205	2.701816359
EBG00000994480	tRNA	0	63.69344	0	390.8768	6.136846	2.617497398
EBG00000994512	tRNA	146.9879	37.43584	702.3888	223.2288	5.962969	2.576030938
EBG00000994453	tRNA	136.4651	34.75582	630.0117	200.2263	5.760944	2.526305253
EBG00000994489	tRNA	129.7511	33.04585	573.2028	182.1717	5.512696	2.46275812
EBG00000994619	tRNA	141.9157	36.144	581.0482	184.6651	5.109149	2.353082998
EBG00000994554	tRNA	71.07042	18.10067	273.4507	86.90638	4.801279	2.263418674
EBG00000994581	Ms_IGR-7	157.8319	40.19766	586.2521	186.3189	4.635069	2.212590939
EBG00000994477	tRNA	98.6338	25.1207	356.5693	113.3226	4.511126	2.17348754
EBG00000994638	Ms_IGR-7	192.7161	49.08218	683.839	217.3334	4.427949	2.146638589
EBG00000994468	tRNA	28.96397	7.37673	100.1194	31.81933	4.313473	2.10884994
EBG00000994546	tRNA	0	22.36695	0	96.10215	4.296613	2.103199973
EBG00000994484	tRNA	80.5	20.50226	270.1622	85.86124	4.187891	2.066224008

### Rv1509 expression in *Mycobacterium smegmatis* also resulted in the downregulation of gene regulators and translational genes

Analysis of RNA-seq data revealed that 83 genes were downregulated in Ms_Rv1509 compared to Ms_Vc (cutoff -log2). Most of the downregulated genes were hypothetical genes whose functions have been predicted based on *in-silico* data (Table 4). The gene MSMEG_3022 (a transglycosylase-associated protein) was significantly downregulated (nearly 6-fold) in Ms_Rv1509 compared to the vector control. Such proteins are known to play a role in septa formation during bacterial cell division (Jorgenson et al., 2014). This supports our SEM data, which showed incomplete septa formation in Ms_Rv1509 compared to the respective control, leading to an increased cell length. MSMEG_3141, a conserved domain protein whose *M. tb* ortholog Rv1473a is predicted to be a transcriptional regulator, showed approximately 5-fold downregulation in Ms_Rv1509. MSMEG_0586 and MSMEG_1777, *M. tb* orthologs of Rv0516, and Rv3288 were also downregulated in Ms_Rv1509, which belong to the class of anti-anti Sigma factors. The quantity of EspA, a virulence-related substrate of the type VII ESX-1 secretion system, is regulated by phosphorylation of Rv0516c in *M. tb*. (Hatzios et al., 2013). This protein is essential for the survival of *M. tb* in stress conditions (Garces et al., 2010).

**Table 4:** A list of downregulated genes in Ms_1509 as compared to Ms_Vc by RNA-seq analysis.

	**Ms_Vc**		**Ms_Rv1509**					
**Gene ID**	**Coverage**	**FPKM**	**Coverage**	**FPKM**	**Fold change**	**Log2 fold change**	**Gene description**	**Presence in Mtb and function**
MSMEG_1519	7,396.581	2,057.059	377.1757	119.8716	−17.2	−4.08974	infA translation initiation factor IF−1	Rv3462c
MSMEG_1777	9,966.036	2,808.248	381.3754	136.1707	−20.64	−4.35614	UsfY protein	Rv3288c Anti anti sigma factor
MSMEG_0586	18,203.45	5,328.442	509.8564	169.5264	−31.52	−4.96592	Stas domai, putaive	Rv0516c Anti-anti-sigma factor
MSMEG_6467	14,132.98	3,661.109	332.0109	110.4988	−33.28	−5.03757	Starvation-induced DNA protecting protein	Absent in Mtb DNA binding
MSMEG_3141	28,966.79	7,583.247	656.1065	210.2984	−36.1	−5.16565	Conserved domain protein	Rv1473A Transcriptional mechanism
MSMEG_3022	27,341.28	7,054.566	328.9183	104.5347	−67.89	−6.06297	Transglycosylase associated protein	Absent in Mtb Septa formation

### Rv1509 enhances the survival of *M. smegmatis* inside RAW264.7 macrophages

**Figure 3:**
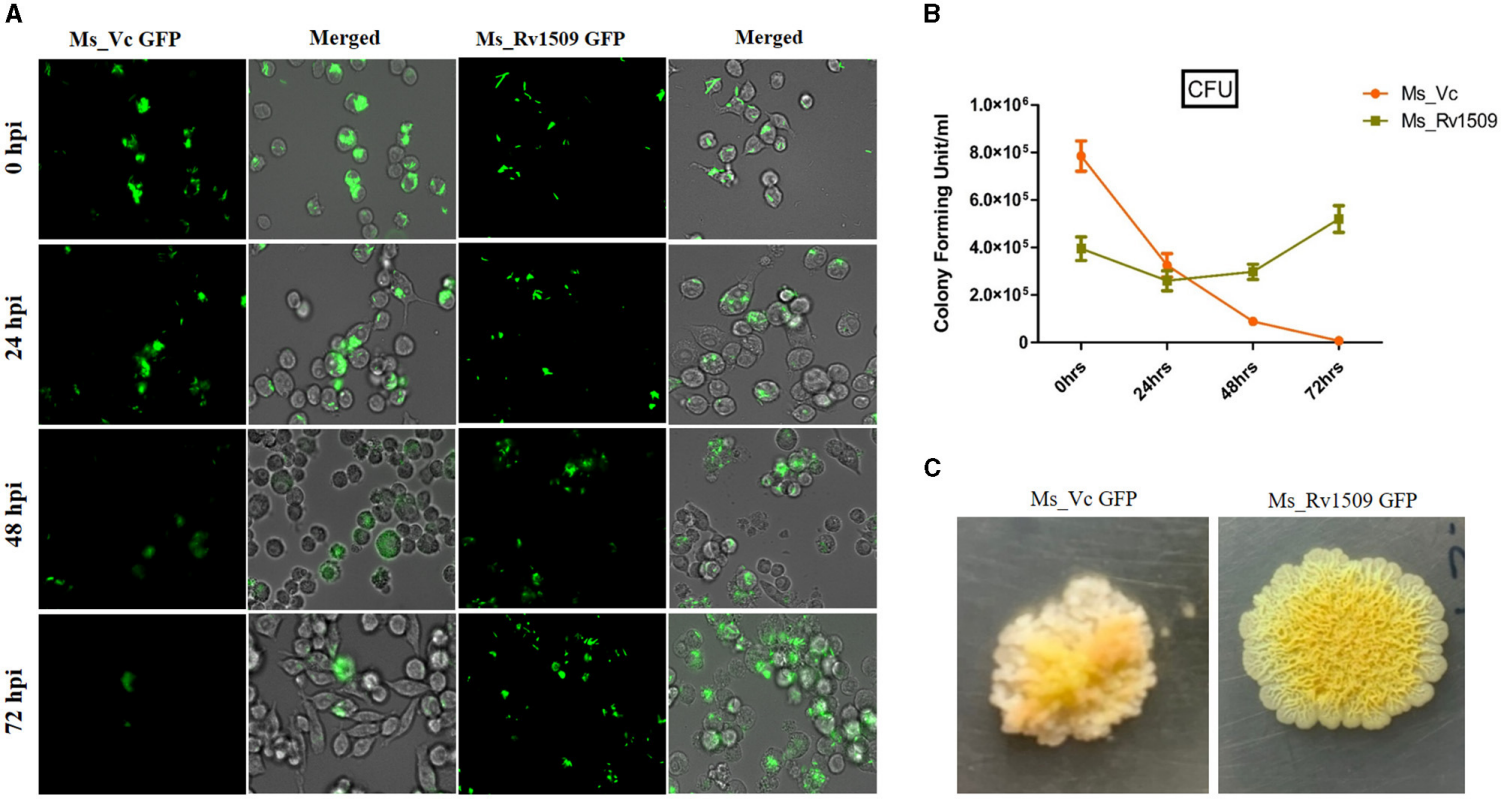
Ms_Rv1509 shows lower uptake and augmented survival compared to Ms_Vc. **(A)** RAW264.7 macrophages were infected with Ms_Vc-GFP and Ms_Rv1509-GFP at an MOI of 10. Cells were monitored at 0, 24, 48, and 72 hpi. **(B)** A bar graph showing the colony forming unit (CFU) of MS_Vc and Ms_Rv1509 following infection in RAW macrophages at different time intervals. **(C)** The colony morphology of Ms_Rv1509 was strikingly different than that of Ms_Vc after the infected RAW cells were lysed and plated on 7H10 agar plates (40X magnification). Fluorescent images were acquired using the EVOS FLauto2.0 microscope (40X magnification) (Thermofisher Scientific). This experiment was repeated three times.

To study the role of Rv1509 protein in survivability and infectivity, murine macrophages (RAW264.7) were infected with GFP-expressing Ms_Rv1509 or Ms_Vc at an MOI of 1:10. Fluorescence microscopic analysis disclosed that there was a significantly reduced uptake of Ms_Rv1509 (Figure 3A) as compared to that of the Ms_Vc within the macrophages. One possible explanation for the low infectivity of Ms_Rv1509 could be attributed to the increased cell length of Ms_Rv1509 compared to Ms_Vc. Interestingly, despite lower uptake of the recombinant *M. smegmatis*, Ms_Rv1509 showed an enhanced survival inside the RAW264.7 macrophages as compared to the Ms_Vc at 72 h post-infection (hpi) (Figures 3A, B). This observation points toward an important role of Rv1509 in bacterial persistence within the host cells. Infection of RAW264.7 cells with Ms_Rv1509 augmented the mycobacterial survival inside the host macrophages, as compared to cells infected with the vector control. This was further validated by colony formation unit (CFU) assay. In addition, the colonies of *M. smegmatis* from infected macrophages showed a different morphology than colonies plated from cells infected with Ms_Vc (Figure 3C). The colonies of Ms_Rv1509 were comparatively smoother and round with translucent edges than that of Ms_Vc, which exhibited dry, rough, and rugose morphology.

### Enhanced survivability of Ms_1509 was due to suppression of phagolysosomal maturation and nitric oxide expression levels in cells

**Figure 4:**
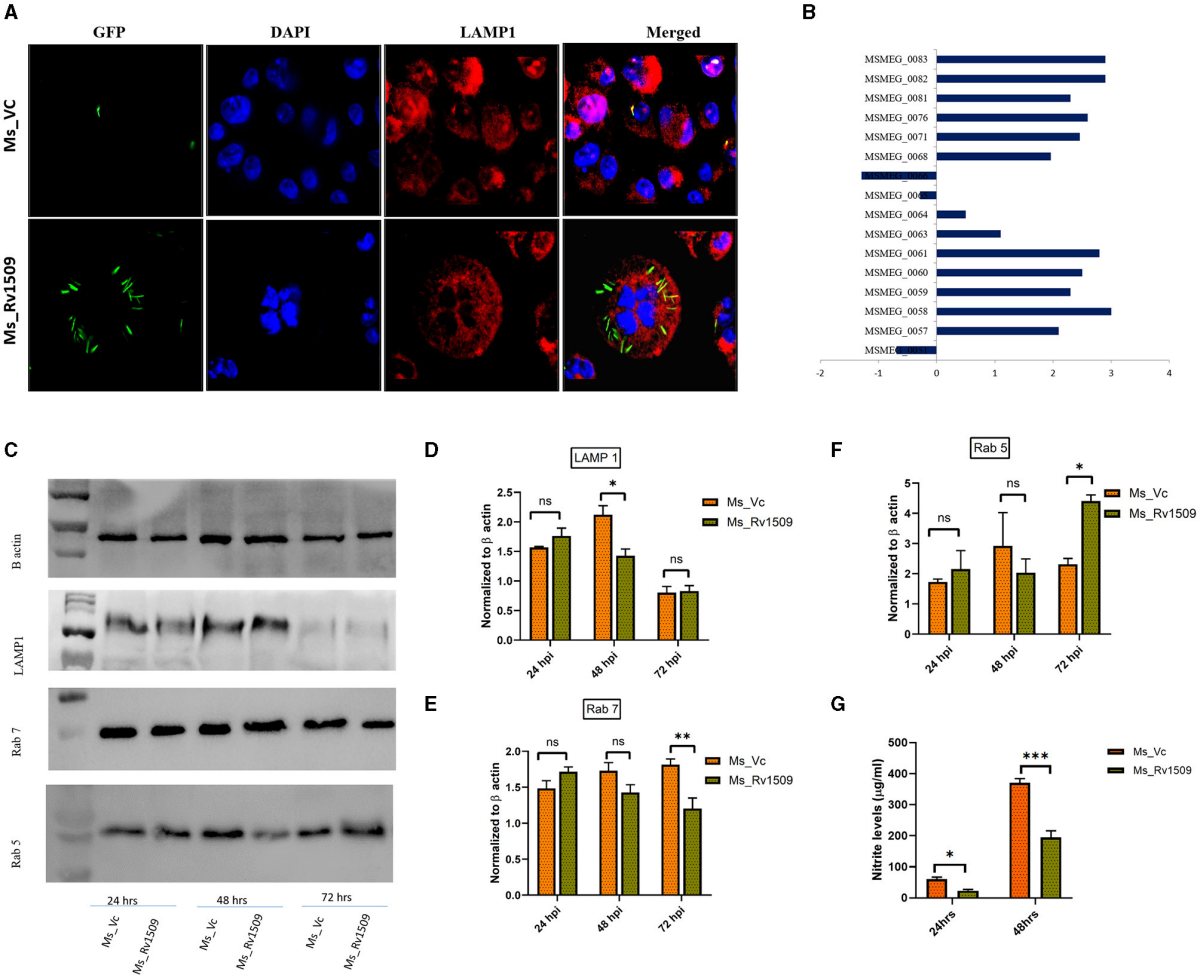
Ms_Rv1509 escapes phagolysosomal maturation for its survival. **(A)** Fluorescent microscopic images of RAW 264.7 cells infected with either Ms_Rv1509_GFP or Ms_Vc_GFP showing levels of phagolysomal maturation (Green (GFP)-Bacteria, Red-Lysosomal marker (LAMP1) and DAPI (Blue)-Nucleus of Host cells). Bacteria inside the phagolysosome appear yellow, whereas bacteria escaping phagolysosomal maturation appear green in color (magnification 100X). **(B)** The Box plot shows the differential expression of genes of the ESX-1 secretion system from Ms_Rv1509. **(C)** Western blots show the expression of LAMP1, Rab7, and Rab5 along with β-actin. **(D–G)** Host macrophages infected with Ms_Rv1509 showed significantly lower levels of LAMP1 (48 hpi), lower levels of Rab7 (Late endosomal marker (72 hpi), and higher levels of Rab5 (early endosomal marker, 72 hpi) as compared to the cells infected with Ms_Vc. **(G)** Cell culture supernatants were assessed for NO levels using Griess reagent assay 24 and 48 hpi of RAW macrophages infected with Ms_Rv1509 or Ms_Vc (*P* ≤ 0.05 =^*^, *P* ≤ 0.01 =^**^ and *P* ≤ 0.001= ***). All the experiments were repeated three times.

A major well-known survival strategy of *M. tb* inside macrophages is mediated by inhibiting the phagolysosomal maturation inside the host cells and escaping into the cytosol. Since Rv1509 knock-in, *M. smegmatis* survives longer than the vector control within the host macrophages, we examined the role of Rv1509 in phagolysosomal maturation inside the host macrophages. Fluorescence microscopic studies in RAW 264.7 cells infected with Ms_Rv1509_GFP revealed inhibition of phagolysosomal maturation contrary to that of Ms_Vc_GFP (Figure 4A). Analysis of RNA-seq data revealed that the ESX I secretion system of Ms_Rv1509 was upregulated, which in turn supports our findings of phagolysosomal escape (Figure 4B). Western blot analysis of host macrophages infected with Ms_Rv1509 showed a significant reduction of Rab7 (late endosomal marker) expression levels and an increased expression of Rab5 protein levels (early endosomal marker) as compared to cells infected with Ms_Vc 72 hpi. There was a significant difference in LAMP1 expression at 48 hpi but no change at 72 hpi (Figures 4C–E). Further, we investigated the role of Rv1509 in regulating nitric oxide (NO) levels inside macrophages. It is a key antimycobacterial molecule and has a crucial role in regulating cellular signaling and innate immune responses during mycobacterial persistence within the host cells. RAW264.7 cells infected with Ms_Rv1509 exhibited reduced NO expression levels at both 24 and 48 hpi (Figure 4F) compared to cells infected with Ms_Vc suggesting a weak bactericidal effect of NO in the presence of the Rv1509 gene. Increasing lines of evidence have shown that attenuation of NO expression levels, at least in part, is responsible for mycobacterial persistence within the macrophages (Mishra et al., 2013). Since NO and its by-products suppress mycobacterial growth, it is possible that the bacteria recruit its own factors to inhibit the production of NO during infection. These results suggest that recombinant *M. smegmatis* expressing Rv1509 protein escapes the maturation of the phagolysosome complex and dampens NO production, thereby exhibiting an augmented survival inside the host macrophages.

### Macrophages infected with Ms_Rv1509 promote necrosis to promote bacterial replication and dissemination

**Figure 5:**
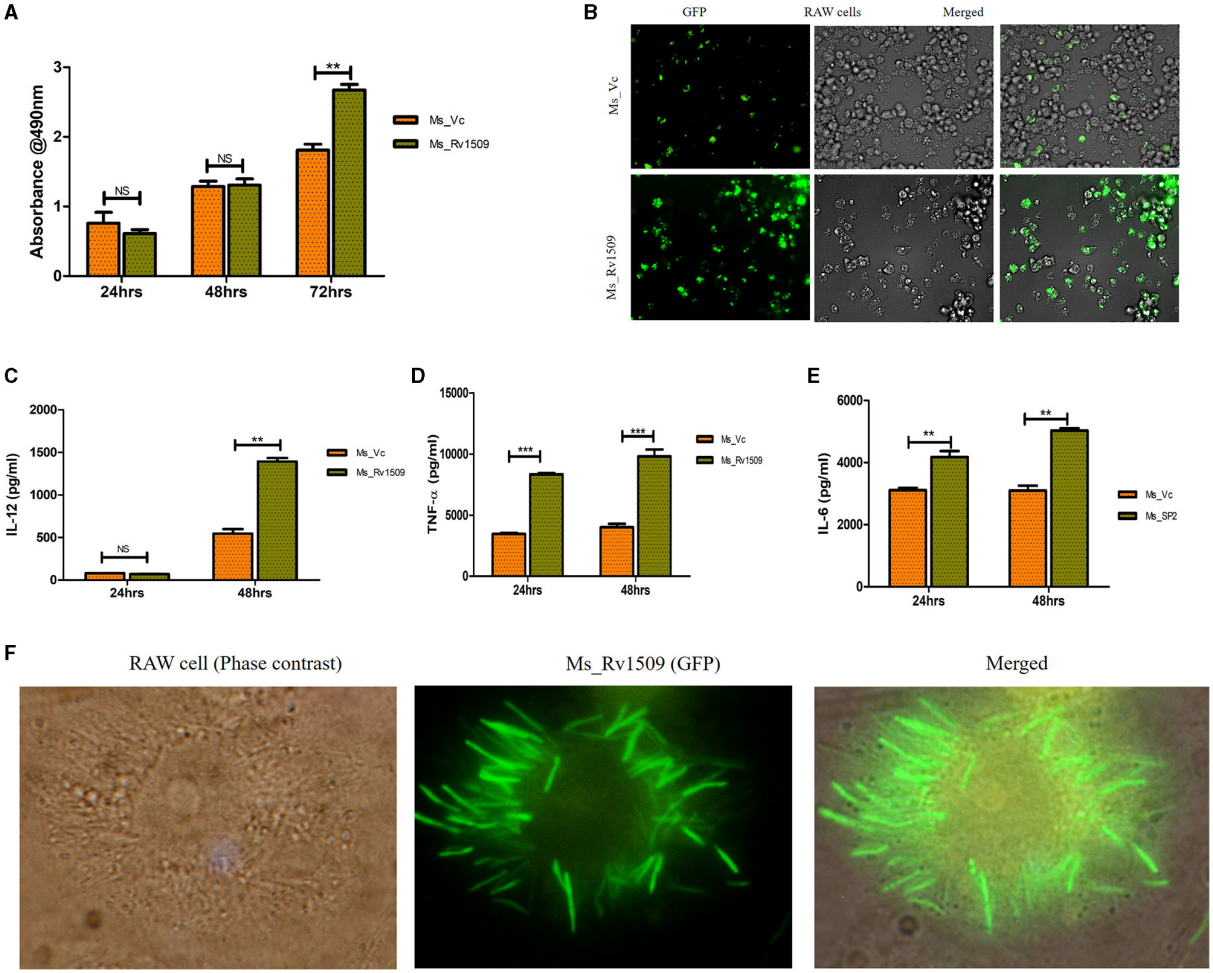
Necrotic mode of cell death in infected macrophages and augmented survival of Ms_Rv1509. **(A, B)** Necrosis was assessed by measuring secreted LDH levels in cells infected with Ms_Vc or Ms_Rv1509 separately at 24, 48, and 72 hpi. RAW 264.7 macrophages were infected with Ms_Vc or Ms_Rv1509 separately. Culture supernatants were collected, and levels of **(C)** IL-12, TNF-α **(D)**, and IL 6 **(E)** were measured using ELISA. **(F)** Fluorescent images showing Ms_Rv1509 replication inside RAW macrophages (72 hpi) [100X magnification (cropped)]. (*p* ≤ 0.01 =^**^, and *p* ≤ 0.001= ***). Fluorescent images were acquired using the EVOS FLauto2.0 microscope (Thermofisher Scientific). Experiments were repeated three times.

Necrosis is a mechanism known to benefit the survival of *M. tb* inside macrophages (Roca et al., 2019). During infection of macrophages with Ms_Rv1509, an increase in visible cell death was observed. To determine whether this cell death was due to necrosis or apoptosis, we performed a Lactate Dehydrogenase assay (LDH assay). The necrotic mode of cell death was evident upon measurement of LDH levels in cells infected with Ms_Rv1509. Cells infected with Ms_Rv1509 exhibited an increase in LDH levels as compared to cells infected with the vector alone (Figure 5A). These results point toward the role of Rv1509 in triggering necrosis and thus promoting mycobacterial dissemination and survival (Figure 5B). Moreover, tumor necrosis factor-alpha (TNF-α) is a critical component of the innate host defense system and plays a crucial role in mediating necrosis (Roca et al., 2019). To analyze the association between TNF- α levels and necrosis, we measured the levels of TNF-α from infected macrophages and found a significant increase in levels of secreted TNF-α. An increase in IL-12 (48 hpi) and IL-6 levels were also seen in comparison to Ms_Vc. Significantly high levels of TNF-α were secreted by macrophages infected with Ms_Rv1509 (Figures 5C, E). This suggests that the expression of Rv1509 protein in *M. smegmatis* promotes higher bacterial replication and dissemination in cells infected with Ms_Rv1509, as compared to cells infected with Ms_Vc. This was evident from the presence of an exceptionally larger number of bacilli in a single infected macrophage (Figure 5F). Further, RAW 264.7 cells infected with Ms_Rv1509 showed an unusually enlarged cell size as well as an enlarged nucleus (Supplementary Figure S4) compared to cells infected with Ms_Vc. After 72 hpi, the recombinant *M. smegmatis* expressing Rv1509 disseminates and spreads the infection to the uninfected macrophages, whereas cells infected with vector control are cleared by the host. These data point to an important role of Rv1509 protein in bacterial virulence and pathogenicity.

### Multinucleated giant cell-like structures are formed among macrophages in response to infection with Ms_Rv1509

**Figure 6:**
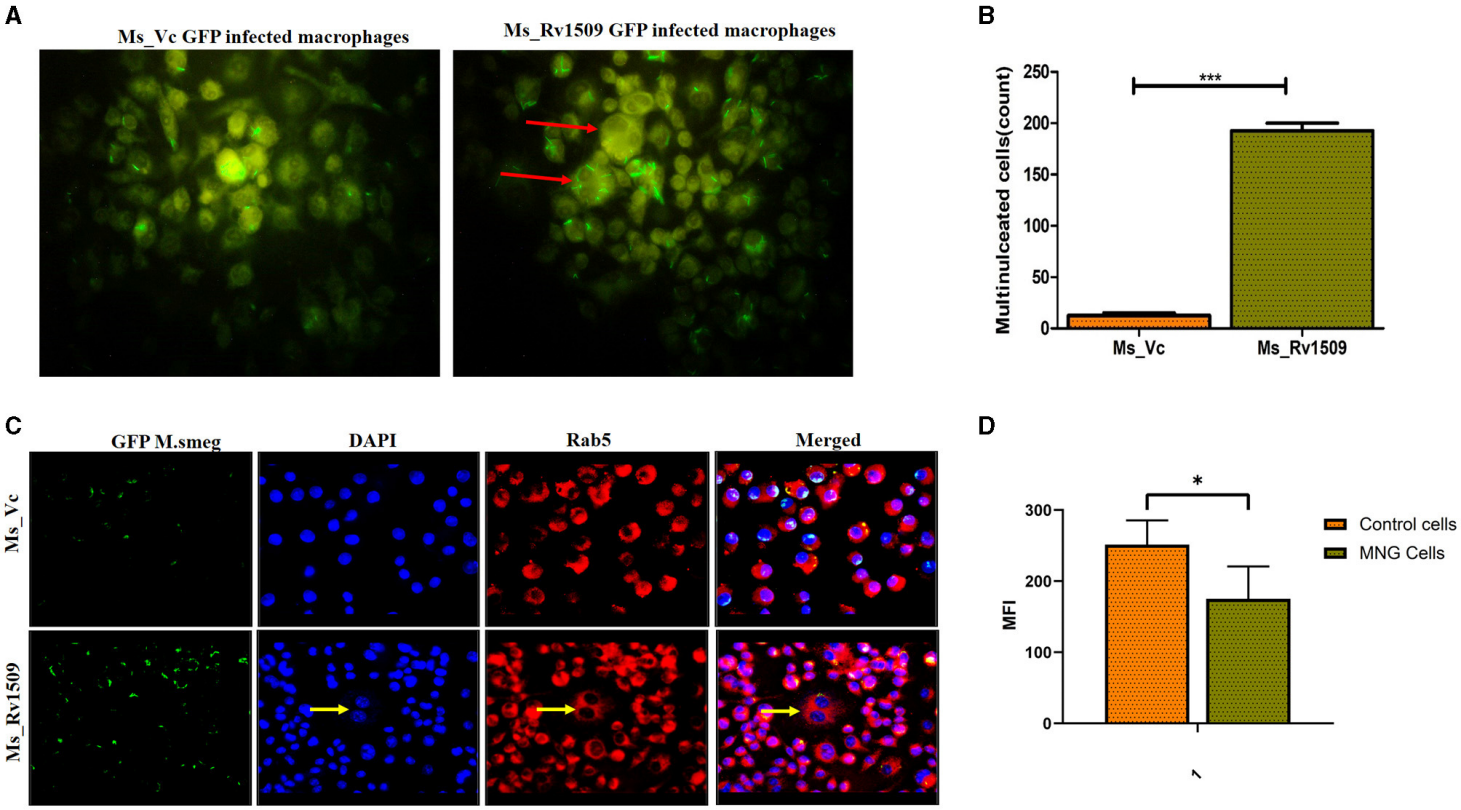
Multinucleated giant cell formation in macrophages (RAW 264.7) after infection with Ms_Rv1509 infection. **(A)** Fluorescent microscopic images show the formation of multinucleated giant cell-like structures in response to infection with Ms_Rv1509 (magnification 100X). **(B)** A bar graph showing the number of multinucleated giant cells present in 10,000 infected macrophages. **(C)** Fluorescent images (magnification 40X and the scale bar represents 20 μm) showing the expression pattern of Rab5 in multinucleated giant cells, which is significantly lower than the uninfected macrophages (GFP-Green-Bacteria, DAPI-Blue-Nucleus, and Red- Rab5). **(D)** The mean fluorescence intensity of control cells vs multinucleated giant cells. (*p* ≤ 0.05 =^*^ and *p* ≤ 0.001= ***). Experiments were repeated three times.

It is well-known that the monocyte/macrophages are capable of fusing with each other to form multinucleated giant cells during infection with *M. tb* (Pegoraro et al., 2014). However, their recognition, fusion, and activation signaling pathways are not completely understood. Macrophages fuse together to form multinucleated giant cells (MGC) in granuloma bodies, which are associated with various pathological conditions following HIV, tuberculosis, and herpes infection (Stockton and Torres, 2020). Interestingly, we also observed multinucleated giant cell-like structures in macrophage cultures infected with Ms_Rv1509. Live cell imaging of the macrophages infected with Ms_Rv1509 demonstrated swift movement of the macrophages from one place to another as compared to Ms_Vc. After 72 h of infection, the formation of giant cells was observed, and the number of total multinucleated giant cells was assessed microscopically (Figure 6A) and estimated to be more than 250/10,000 macrophages in Ms_Rv1509 compared to 20/10,000 in the case of Ms_Vc (Figure 6B). Thereafter, Rab5 antibody and DAPI were used to visualize giant cells *in vitro*. The giant cells expressed low levels of specific markers Rab5 (early endosomal marker) and low staining of DAPI (nucleus) (Figure 6C). These results further complemented the analysis of the mean fluorescence intensity of MGC and normal cells (Figure 6D). Therefore, it is likely that infection with Ms_Rv1509 attracts individual macrophages to fuse together to form giant cells.

### Enhanced survival of Ms_Rv1509 in various organs of infected mice

**Figure 7:**
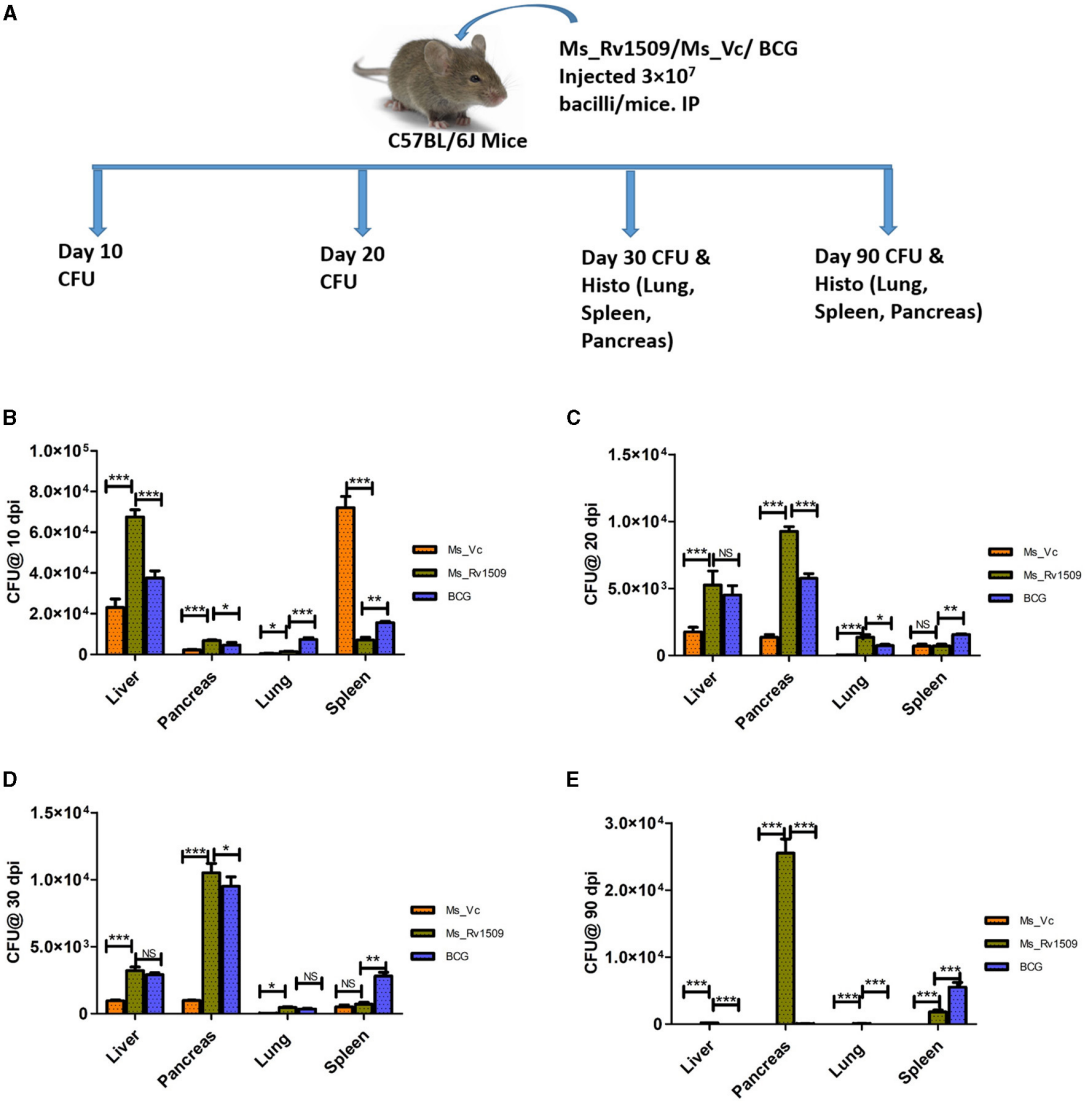
Differential survival of Ms_Vc, Ms_Rv1509, and BCG in various organs of infected mice. **(A)** Schematic representation of mice experimental design. **(B)** Presence of bacilli in C57BL/6 mice infected mice with Ms_Vc, Ms_Rv1509, and BCG at 10 dpi. **(C–E)** Colony forming unit of Ms_Vc, Ms_Rv1509, and BCG from pancreas, liver, lung, and spleen of C57BL/6 mice 20, 30, and 90 days post-infection (*p* ≤ 0.05 =^*^, *p* ≤ 0.01 =^**^, and *p* ≤ 0.001= ***).

To further gain insights into the role of Rv1509 in virulence and pathogenesis, three groups of C57BL/6 mice were infected with Ms_Vc, Ms_Rv1509, and *M. bovis*_BCG respectively, through the intra-peritoneal route with a dose of 3 × 10^7^ bacilli/mice_(Figure 7A). Initially, 3 mice per group were infected to standardize the experiment and sacrificed at days 10 and 20 post-infection. Initial results revealed that the number of Ms_Rv1509 and *M. bovis*_BCG was significantly higher in the liver, pancreas, and lung, whereas Ms_Vc was found higher in only spleen cells 10 days post-infection (Figures 7B, C). These preliminary findings further prompted us to check for the persistence of Ms_Rv1509 beyond 20 days. Thereafter, we infected 5 mice per group for a duration of 30 days and 90 days via the intraperitoneal route. Mice were sacrificed, and lungs, liver, spleen, and pancreas were collected and harvested for the analysis of bacterial burden at both time points. A macerated sample (10 μl) was used to make smears for AFB staining to visualize the bacteria. Our results demonstrated that Ms_Rv1509 and *M. bovis*_BCG were detected in the spleen, liver, lung, and pancreas, whereas Ms_Vc was detected only in the spleen, liver, and pancreas 30 days post-infection, albeit at much lower levels (Figures 7D, E). Interestingly, after 90 days of infection, Ms_Rv1509 bacterial load had increased further in the pancreas, but no BCG or Ms_Vc was detected, with the exception of BCG in the spleen.

### Multinucleated giant cells and granuloma-like structures formation in the pancreas of mice infected with Ms_Rv1509

**Figure 8:**
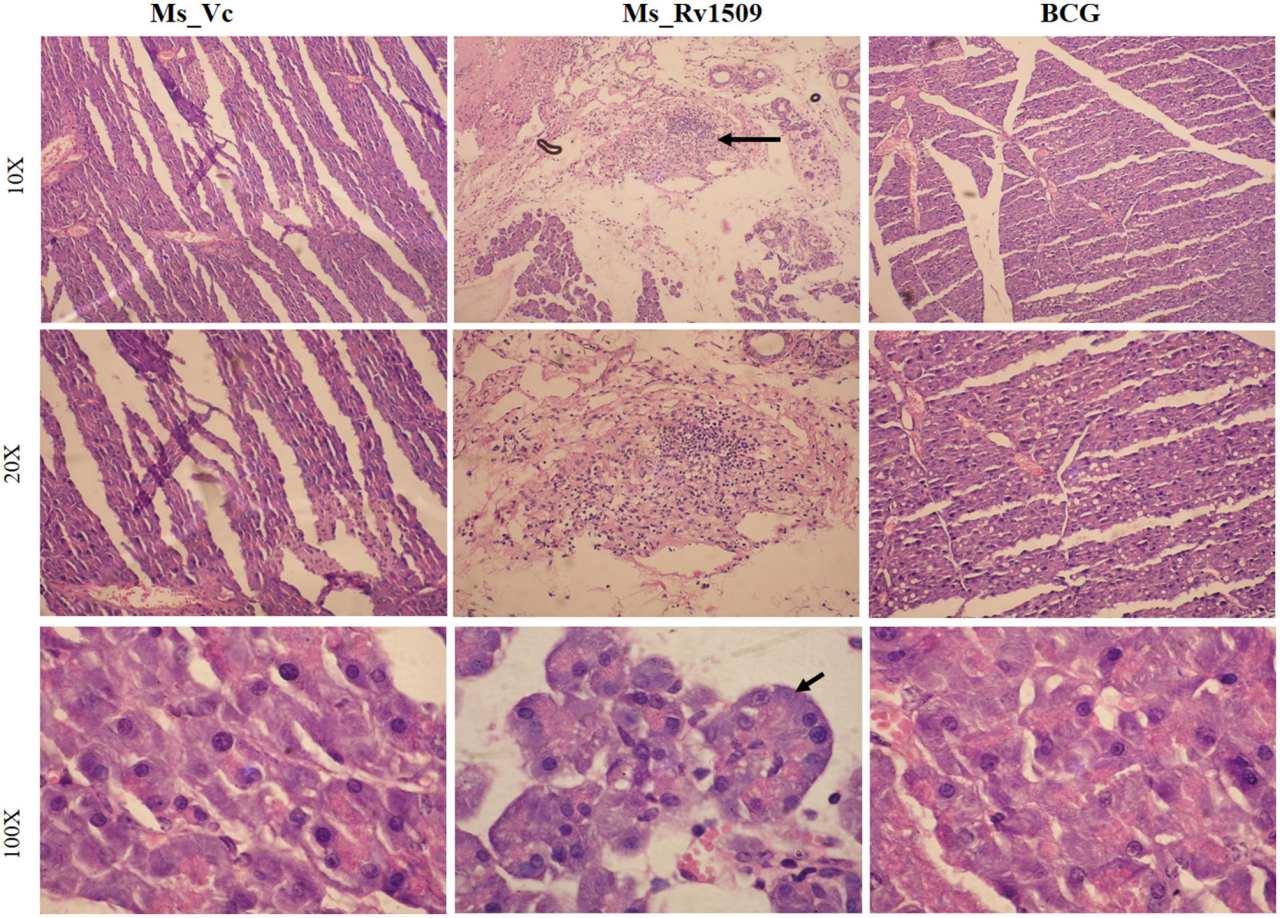
Histopathology of C57BL/6J mice pancreatic tissues. HandE (Hematoxylin- Blue- Nucleus and Eosin- Pink- Cytoplasm) stained pictures of Ms_Vc, Ms_Rv1509, and BCG infected mice pancreatic tissue at day 90 post-infection (row 1) (10X magnification). Lymphocyte migration and multinucleated cells in Ms_Rv1509 infected mice pancreatic tissue. Multinucleated giant cells of Ms_Rv1509 infected mice pancreas at 90 days post-infection (row 2–20X and row 3–100X magnification) in comparison with Ms_Vc and BCG.

Further, histological assessment revealed no alteration in the tissue morphology of organs collected on day 30 (Supplementary Figure S5A). Interestingly, pancreatic tissues harvested on day 90 post-infection showed a lot of lymphocyte degeneration and multinucleated giant cell-like structures in Ms_Rv1509 infected mice (Figure 8). The images with 10X, 20X, and 100X magnification revealed the presence of giant cells and granuloma-like structures at 90 days post-infection (Figure 8). The spleen was enlarged in the mice infected with Ms_Rv1509 in comparison to Ms_Vc and BCG-infected mice (Supplementary Figure S5B). There was no change in the morphology of other tissues like the lung and spleen of Ms_Vc and BCG-infected mice. These results pointed to a regulatory role of Rv1509 in modulating cell-host pathogenesis, tipping toward the pathogen's better survival inside host macrophages.

### Augmented innate and adaptive immune profile in Ms_Rv1509 infected mice

**Figure 9:**
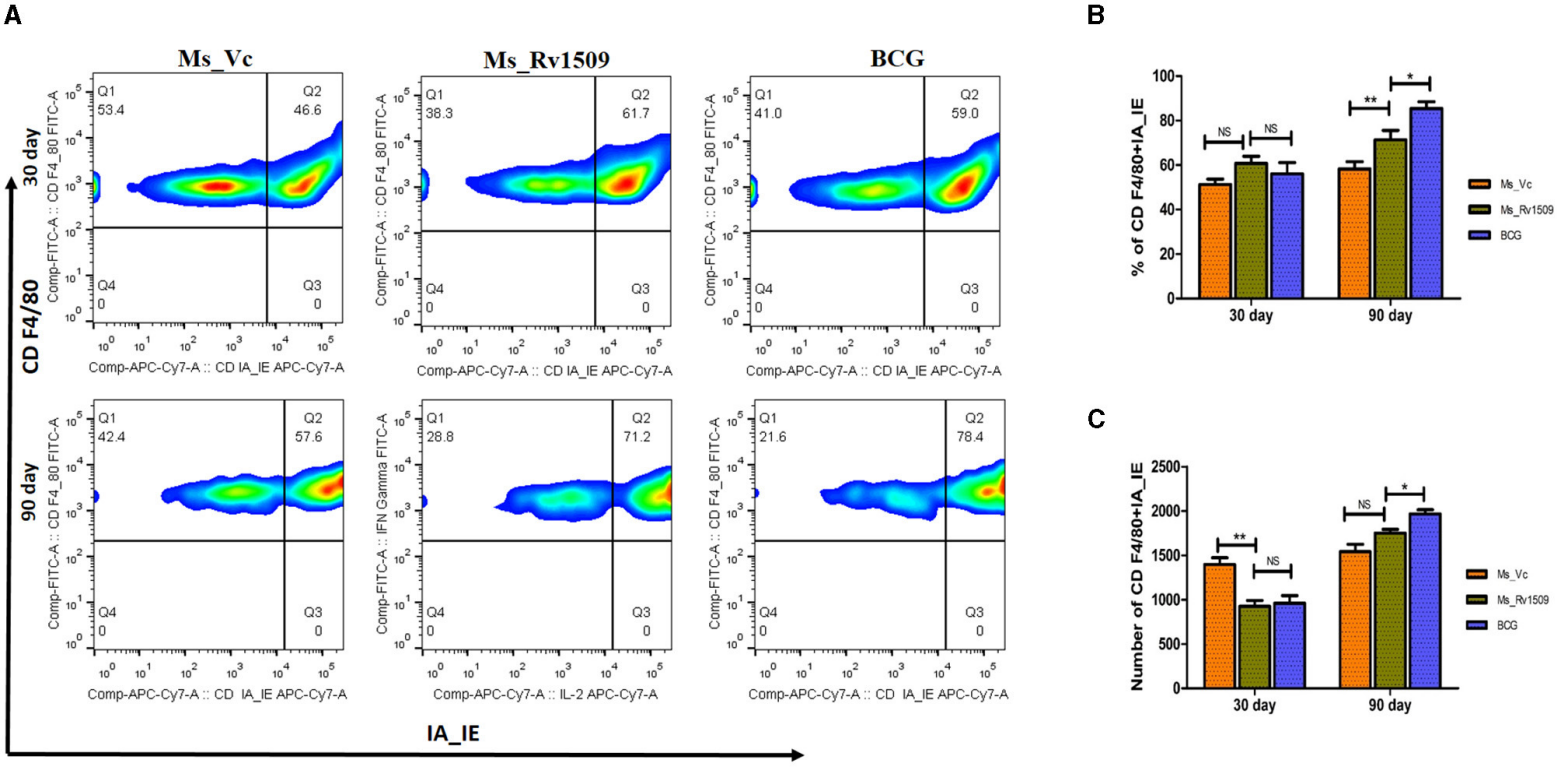
Determination of activation markers on macrophages due to infection with Ms_Vc, Ms_Rv1509, and BCG. **(A)** Representative FACS plot illustrating activation maker CD IA-IE (MHC-I) on macrophages (CD F4/80). The percentage of macrophages (mice splenocytes) expressing activation marker IA-IE was measured by flow cytometry post 30 days **(B)** and 90 days **(C)** infection. (*p* ≤ 0.05 =^*^ and *p* ≤ 0.01 =^**^).

The spleen cells were stained with surface markers of macrophages, such as F4/80 (naive macrophages) and MHC-II -IA/IE, to investigate the effect of Ms_Rv1509 on immune modulation. FACS data analysis 30 days post-infection did not show a significant difference in the number or activatory phenotype of macrophages between Ms_Vc, Ms_Rv1509, and *M. bovis*_BCG (Figures 9A, B). However, after 90 days of infection, the number of activated macrophages in the spleen and peritoneum of Ms_Rv1509 infected mice was significantly higher than Ms_Vc and *M. bovis*_BCG infected mice (Figures 9B, C).

T cells have been known to play a crucial role in maintaining antimycobacterial immunity (10.1038/s41579-022-00763-4). To investigate the T cell responses in mice infected with Ms_Vc, Ms_Rv1509, and BCG, spleen cells were stained with CD3, CD4, CD8, and CD25 surface markers. It was observed that the CD3 cell population in Ms_Rv1509 infected mice significantly increased after 90 days of infection, whereas no change was observed at 30 days post-infection (Supplementary Figures S6A, C, S7B). Likewise, an increased CD8 population was observed in the spleen cells of Ms_Rv1509 infected mice after 90 days of infection, but no change at 30 days post-infection was seen (Supplementary Figures S7A, C, S8B). During the initial stage of infection, an increased number of CD4^+^CD25^+^ cells was observed in Ms_Rv1509 infected mice as compared to Ms_Vc or *M. bovis*_BCG (Supplementary Figure S8A). During long-term survival (90 days), there was no statistical difference between the activated T cells in Ms_Rv1509 and BCG-infected mice, but the levels in both these groups were significantly high compared to Ms_Vc (Supplementary Figures S8B, C), indicating robust activation of T cell response in the presence of Rv1509. Further, we checked the levels of various cytokines such as IL-12, IL-6, IL-1β, TNF-α IFN-γ, IL-4, and IL-10 in the serum of infected mice. There was a significant increase in cytokines such as IL-1β, IL-6, IL-12, and TNF-α (Supplementary Figures S9A–D). A significant difference in IL-1β was observed at 30 days post-infection, but there was no change at 90 days post-infection compared to Ms_Vc. Interestingly, higher IL-6 and TNF-α levels were observed in BCG-infected mice after 90 days of infection as compared to Ms_Vc and Ms_Rv1509. The cytokines IFN-γ, IL-4, and IL-10 were undetectable (data not shown).

## Discussion

Elucidating the function(s) of the “hypothetical” and “uncharacterized” genes of *M. tb* would help us better understand mycobacterial virulence and pathogenesis. We investigated the function of Rv1509, a putative hypothetical protein found only in the *M. tb* complex and absent in other mycobacterial species. *In silico* analyses revealed that the Rv1509 protein has a DNA binding motif and a methyltransferase domain, thus may alter the gene expression pattern. Most of the time, epigenetic mechanisms involve DNA methylation, which is one of the important regulators of gene expression and virulence in mycobacteria. Understanding the interplay between epigenetic mechanisms and bacterial pathogenesis could potentially lead to the development of novel therapeutic strategies for combating mycobacterial infections.

*M. smegmatis* is a known surrogate model organism that delineates the role(s) of uncharacterized *M. tb* proteins. The ease of genetically modifying *M. smegmatis* combined with shorter doubling time and low risk to lab workers makes it an attractive model for the study of various aspects associated with *M. tb* (Ranjitha and Rajan, 2020). Nevertheless, limitations associated with *M. smegmatis*, including non-pathogenicity and non-virulence, need to be taken into consideration while interpreting the data.

We created a knock-in strain of *M. smegmatis* expressing Rv1509 protein using an integrative vector pST-Ki. Interestingly, the expression of the *Rv1509* gene in *M. smegmatis* retarded the growth of *M. smegmatis* by 8 h, with a doubling time of 12 h for the recombinant *M. smegmatis* as compared to the usual doubling time of 3–4 h of wild-type *M. smegmatis*. The knock-in *M. smegmatis* (Ms_Rv1509) showed an increase in cell length (3.5 to 9 μm) as compared to the (Ms_Vc) vector containing *M. smegmatis* (1.5 to 4 μm). RNA-seq-based transcriptomic data revealed several genes to be differentially regulated between Ms_Rv1509 and Ms_Vc. Based on the RNA-seq data (GSE126837), a gene called transglycosylase-associated protein (MSMEG_3022), present in *M. smegmatis*, was dramatically downregulated. The transglycosylase-associated protein family is a well-studied protein known to play a role in septa formation during cell division (Jorgenson et al., 2014). Downregulation of this gene could, in part, play a role in incomplete septa formation in knock-in *M. smegmatis*, subsequently leading to increased cell length. TEM analysis indicated that the ribosomal content was increased in Ms_Rv1509 in the log phase compared with Ms_Vc. Further, 2D-gel electrophoresis indicated a differential expression of proteins between Ms_Rv1509 and Ms_Vc, suggesting that the Rv1509 protein probably regulates transcription and translational machinery to prolong the persistence of mycobacterium inside the host.

The RNA-seq data revealed that genes involved in virulence, pathogenicity, antibiotic resistance, and ABC transport system were differentially regulated. EspR is a known transcriptional regulator that activates the ESX-1 secretion system and controls virulence and pathogenesis in *M. tb* (Raghavan et al., 2008). The expression of Rv1509 induces the expression of EspR orthologs in knock-in *M. smegmatis*, which otherwise do not express it at all. This suggests that Rv1509 possibly regulates the Esx-1 secretion system in *M. tb* and could have profound implications for the pathogenesis of TB. In addition, most of the genes upregulated in our RNA-seq data belong to the class of transcription regulators, pointing to the role of Rv1509 in altering the transcriptional network to facilitate survival inside the host.

Transcriptional regulators such as the MarR family of proteins, which were found to be upregulated, play an important role in multiple antibiotic resistance (Healy et al., 2016). The upregulated GntR family of proteins is known to bind DNA through a helix-turn-helix motif and regulate various biological processes (Zeng et al., 2016). LysR family of proteins, transcriptional regulators that control genes involved in virulence, metabolism, and quorum sensing, are upregulated (Domenech et al., 2001). LuxR and TetR family of proteins are also upregulated, and they are key players in quorum sensing and coordinate the expression of several genes involved in virulence, antibiotic biosynthesis, plasmid transfer, transcriptional repressors, antibiotic resistance and bacterial pathogenesis, and biofilm formation (Srivastava et al., 2017). TetR family of proteins are usually transcriptional repressors, inhibiting the expression of certain genes. These proteins play a role in antibiotic resistance, bacterial pathogenesis, and cell stress (Pushparajan et al., 2020). It is likely that Rv1509 expression, in combination with other *M. tb* proteins, promotes and enhances the survival of the bacteria in response to changing conditions inside the host during the course of infection.

*M. tb* has adopted various strategies to circumvent the hostile conditions inside the host. One of the well-characterized mechanisms is mediated by evasion of phagolysosomal maturation, subsequently leading to inhibition of antigen presentation to T cells for generating an effective immune response. Intriguingly, the knock-in Ms_Rv1509 escaped entering into phagolysosomes compared to the Ms_Vc, as seen by Rab7 protein levels in our Western blot analysis. Emerging evidence has shown that virulent *M. tb* inside phagosomes escape acquiring late endosome marker Rab7, thereby preventing phagosome maturation (Padhi et al., 2019). Rab5 and Rab7 facilitate early and late endosome fusion, respectively. Our results demonstrated that Ms_Rv1509 escaped phagosomal maturation, thereby translocating to the cytoplasm and thus persisting inside the macrophages for an extended time. As ESX-1 has been implicated in the phagolysomal escape in *M. tb* (Wong, 2017), it is interesting to note that Rv1509 expression in *M. smegmatis* imparts a virulent phenotype to a non-pathogenic bacterium. This corroborated the earlier observation regarding the expression of EspR orthologs in Ms_Rv1509. It would be interesting to delineate the corresponding secretion machinery in *M. smegmatis* to better understand the type VI secretion system and its role in the pathomechanism of TB disease.

To investigate other factors responsible for the increased survival of Ms_Rv1509 in macrophages, we measured the levels of NO, which is a key host bactericidal molecule against *M. tb* (Nieto-Patlan et al., 2019). We found that NO levels were downregulated in macrophages infected with Ms_Rv1509 as compared to cells infected with Ms_Vc 24 and 48 hpi. NO is an intracellular messenger that has been recognized as one of the most versatile in the immune system. It also regulates the functional activity of immune cells. NO and RNI kill intracellular pathogens, including mycobacteria, with levels correlating with the antimycobacterial defense. Low levels of NO lead to better pathogen survival by low bactericidal activity (Yang et al., 2009; Bhat et al., 2013).

Next, to assess the immuno-modulatory potential of Rv1509, the levels of pro-inflammatory cytokines in infected macrophages were estimated. The levels of secreted TNF-α were upregulated in cells infected with Ms_Rv1509 as compared to cells infected with Ms_Vc 48 hpi. Previous studies have shown that elevated levels of TNF-α mediated necrosis are pivotal to establishing *M. tb* virulence and persistence within the host macrophages (Roca et al., 2019). Consistent with previous findings, our study showed enhanced levels of Ms_Rv1509 induced TNF-α levels in the supernatant of macrophages as compared to the Ms_Vc infected cells, subsequently promoting the survival of Ms_Rv1509 inside macrophages. Though the exact mechanism of TNF-α mediated necrosis was not divulged, it can contribute to cell necrosis through multiple mechanisms, including induction of cell death by extrinsic apoptosis pathway or necroptosis mediated by specific signaling pathways. TNF-α also promotes the recruitment and activation of immune cells and the release of other pro-inflammatory cytokines to activate cell death pathways. The Ms_Rv1509-induced necrosis correlated with higher levels of secreted LDH in macrophages, directly implicating enhanced survival and spread. Moreover, the persistence of *M. tb* inside macrophages induces the recruitment of cells, including lymphocytes, DCs, and macrophages at the infectious site, followed by the fusion of macrophages to form multinucleated giant cells (MGCs) (Mezouar et al., 2019). These granulomatous structures accumulate viable bacteria inside, leading to a cascade of signaling events (Lay et al., 2007). Interestingly, confocal microscopy revealed MGC-like structures in cells infected with Ms_Rv1509 as compared to the macrophages infected with the vector control. The multinucleated cells included nuclei ranging from 3 to 5 in number. In rare cases, the formation of granulomatous-like structures in cells infected with non-pathogenic mycobacteria such as *M. smegmatis* has been demonstrated earlier (Alqurashi et al., 2019; Dos Santos et al., 2019). Differentiation of macrophages to form “foam cells” is a hallmark of the lung granuloma structures seen in active tuberculosis patients. Restructuring of cell wall hydrophobicity and lipids increases the capacity of macrophages to form granuloma structures (Vermeulen et al., 2017).

*M. smegmatis*, which usually gets cleared in C57BL/6 mice in 15–20 days (Bange et al., 1999), was able to survive for more than 90 days with ectopic expression of Rv1509. After 90 days of infection, Ms_Rv1509 and BCG bacteria were observed to survive in the liver, pancreas, spleen, and lungs, whereas Ms_Vc cleared in 20 days post-infection. Histological examination of the pancreas tissue revealed extensive lymphocyte migration, giant cell formation, and granuloma-like organizations. Generally, giant cell formation and granuloma can be seen in *M. tb* infection to clear the pathogen. There are no such reports that *M. smegmatis* can cause pathological conditions in mice infection studies. It was intriguing to observe that ectopic expression of just a single protein from *M. tb* could instigate virulent characteristics in a well-characterized non-pathogenic mycobacterial species. Further, the excessive bacterial load of Ms_Rv1509, preferably in the pancreas, was astonishing. More exploratory studies would be required to delineate this divergence in a niche in place of lungs as observed in normal *M. tb*. In our study, though macrophages were activated in mice infected with Ms_Rv1509 by enhancing the pro-inflammatory cytokines, there was no bacterial killing. Optimal activation of macrophages in *M. tb* infection is critical in pathogen clearance (Park et al., 2019). It was intriguing that activated immune cells were unable to clear the infection in the pancreatic tissue. However, these immune cells seem to help in the formation of granuloma-like structures that inhibit pathogen exposure.

**Figure 10:**
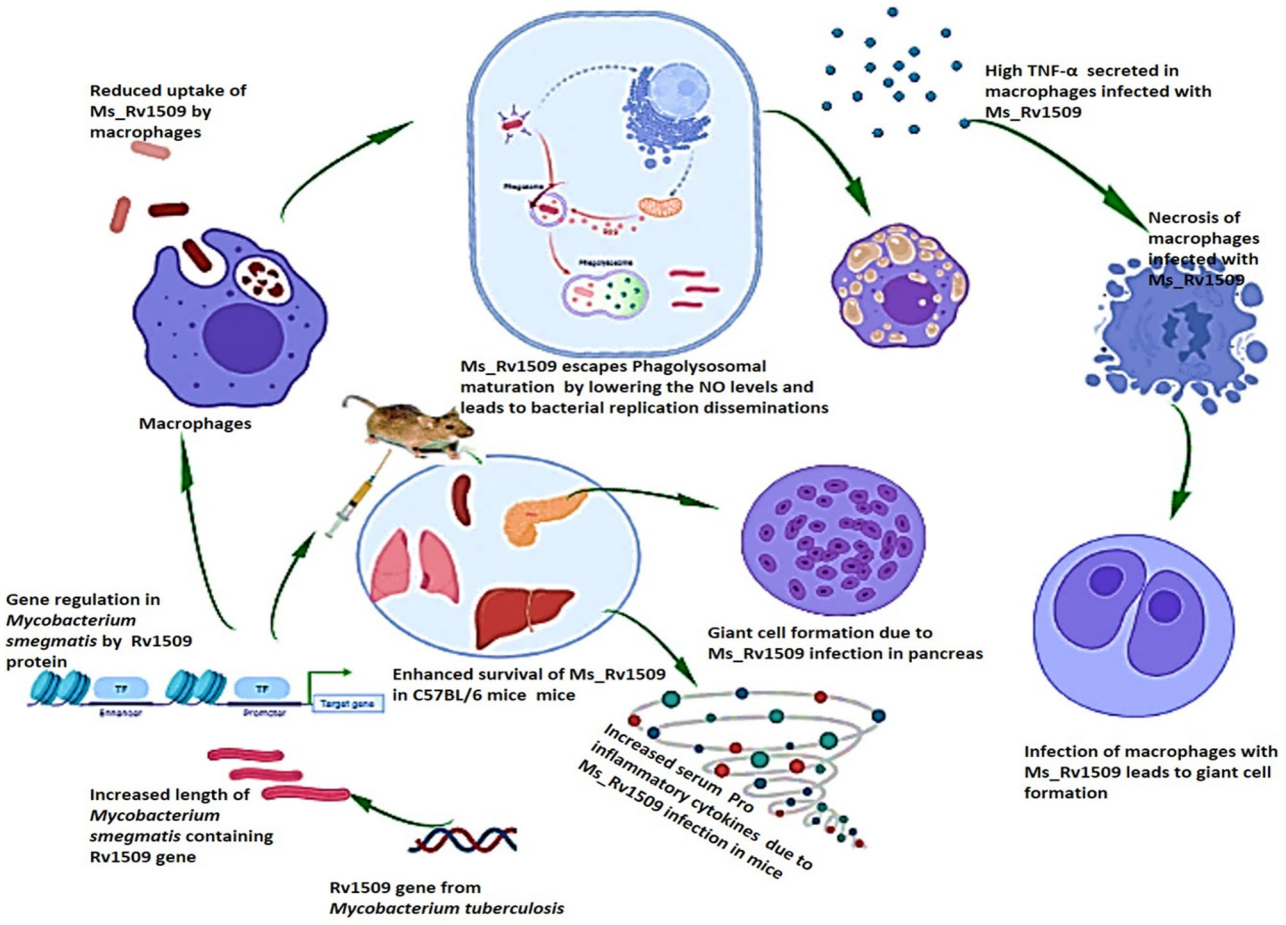
Pictorial summary of the observed effects that explain the role of *M. tb* signature protein Rv1509. Ectopic expression of the *Rv1509* gene in *Mycobacterium smegmatis* leads to altered gene expression of multiple genes, suggestive of it being a global gene regulator. The genes involved in septa formation are downregulated and consequently manifest as an increase in the length of bacteria as well as reduced uptake in macrophages. Ms_Rv1509 could manage to escape from phagolysosomal maturation, which augments its intracellular survival. Macrophages infected with Ms_Rv1509 showed higher production of inflammatory cytokines and were also observed to undergo necrosis. In the mice model, Ms_Rv1509 infection displayed enhanced survival as compared to BCG and Ms_Vc, along with the formation of multinucleated giant cells and granuloma-like structures that demonstrate pathogenic attributes.

Based on our results, we concluded that Rv1509, similar to another MTase, is a master regulator that controls the expression of other important genes playing roles in pathogen virulence. The altered gene expression pattern in Ms_Rv1509 leads to augmented survival under *in-vitro* and *in-vivo* conditions, which further leads to giant cell formation, normally seen in pathogenic bacterial infections. The altered gene expression also results in immune modulation in mice, which is depicted by enhanced activation of immune cells and increased pro-inflammatory cytokines in comparison to Ms_Vc. All the above-mentioned changes in the Ms_Rv1509 are due to ectopic expression of a single gene, which indicates its role in regulating other important genes for the survival of *M. smegmatis* (Figure 10). This multipronged effect of multiple virulent pathways makes this protein an attractive drug target. Moreover, being an *M. tb*-specific protein, it has shown to be a better diagnostic candidate (Quadir et al., 2021), thus marking this protein as an exciting candidate for further explorations for efficient interventions against TB.

### Limitations of the study

While *M. smegmatis* serves as a useful model organism for studying aspects of *M.tb* biology, it is important to acknowledge that there are differences between the two species, particularly in terms of virulence factors, pathogenicity, and specific metabolic pathways. Therefore, while findings from studies using *M. smegmatis* can provide valuable insights into *M.tb* biology, they may not always directly translate to *M. tb* behavior *in vivo*. Nonetheless, *M. smegmatis* remains a valuable tool in tuberculosis research, especially for initial screenings and basic investigations.

## Materials and methods

### Cloning, sub-cloning, expression, and purification of Rv1509

*Rv1509* gene was cloned into a pET-28a(+) vector, expressed, and purified as mentioned previously (Ahmad et al., 2021). The pET-28a_Rv1509 clone and pST-Ki(addgene#44563) were digested with *Bam*H1 and *Hin*dIII (ThermoFisher Scientific) enzymes. The digested pST-Ki and Rv1509 fragments were ligated using the ligation kit (ThermoFisher Scientific, USA) for 30 min. The ligated product was transformed into *E.coli* DH5α cells. The positive clones were selected by colony PCR, and the positive colonies were confirmed using the restriction digestion method (Supplementary Figure S10).

### Rv1509 protein purification

The *E. coli* (Clear Coli) transformed with pET 28a_Rv1509 was grown at 37°C for 2 h using a shaking incubator. Rv1509 protein expression was stimulated by adding 1M IPTG to the culture when OD600 reached 0.2. The culture was further grown for 4 h, and then the bacteria were harvested and sonicated (30 ML PBS+ 0.03% N Lauryl sarcosine). The protein was then bound to the Ni-NTA column (affinity Chromatography) and then washed with 20 mM Imidazole. The protein was eluted using the 300 mM Imidazole and the concentration was checked by Bradford assay.

### DNA-methyltransferase assay

*In silico* studies of Rv1509 protein for the identification of DNA binding motifs were done using the EXPASY Prosite tool (https://prosite.expasy.org/). S-adenosyl methionine (SAM)-dependent DNA methyltransferase activity of recombinant protein Rv1509 was measured using colorimetric EpiQuik^TM^ DNMT Activity/Inhibition Assay Ultra Kit, as per the manufacturer's protocol (110 Bi County Blvd, Ste, 122, Farmingdale, NY 11735). Purified signature protein Rv1509 (1, 2, 4, and 8 μg/ml), substrate, and assay buffer supplemented with SAM were incubated for 120 min at 37°C, followed by washing and addition of capture antibody for 30 min. Wells were washed, and a detection antibody (1:1,000) was added, followed by an enhancer and developing solution (EpiQuik^TM^) for color development. Plates were read at 450 nm with an optional wavelength of 655 nm.

### DNA binding assay

Fluorescence spectroscopy was used to record the protein sample's fluorescence emission spectra. The emission was measured from 310 to 420 nm when the sample was stimulated with 280 nm light. After rapidly mixing, a 1 μl aliquot of DNA (plasmid DNA, 4 kb, 47.41 fmol) was added to the protein solution (300 μg/ml), and the emission spectrum was recorded at various DNA concentrations. In a quartz cuvette, a reaction mixture containing 300 μg/ml protein concentration was created for time-based investigations. A 1 μl aliquot of DNA (plasmid DNA 6 kb, 47.41 fmol) was quickly mixed into the cuvette. The cuvette was immediately inserted in the fluorometer, and time-based readings began. Excitation of the samples was done at 280 nm, and emission was measured at the wavelength of maximal tryptophan (340 nm).

### Creation of knock-in *M. smegmatis* expressing Rv1509 protein

Rv1509-pST-Ki construct was electroporated into *M. smegmatis* (mc^2^155) using an in-house standardized protocol. *M. smegmatis* culture was grown up to O.D_600_ of 0.4 to 0.6, and the bacteria were harvested by centrifugation at 4,500 rpm for 10 min. The pellet was washed with PBS and dissolved in 10% glycerol. All the steps were performed at room temperature (20°C to 25°C). Three consecutive washings were done by gradually reducing the volume of 10% glycerol. The pellet was finally dissolved in 1.0 ml of 10% glycerol, and 200 μl was transferred to an electroporation cuvette (2 mm, Bio-Rad). Plasmid (250 ng/μl) was added to the cells (3 μl) and incubated for 30 min at room temperature. The conditions for electroporation were as follows: Voltage 2,200, Capacitance 25 microns, Resistance 800 Ω, and Cuvette capacity 0.2 mL. Following electroporation, complete media was added to the electroporated cells, followed by incubation at 37° overnight. The next day, cells were plated on 7H10 medium (BD Biosciences) containing 50 μg/mL kanamycin as the selection marker. The colonies grown on the antibiotic plate were used for colony PCR using specific primers for the Rv1509 gene along with positive control (pET28a-Rv1509). The confirmed clones were validated for Rv1509 protein expression.

### Expression of Rv1509 protein in knock-in *Mycobacterium smegmatis*

The Rv1509 gene was knocked in *M. smegmatis* and was grown in 7H9 broth medium with 10% OADC (Hi-Media), 0.5% glycerol, 0.05% Tween 80, and 50 μg/ml Kanamycin. The culture conditions for Ms_Rv1509 GFP or Ms_Vc GFP were also similar to Ms_Vc or Ms_Rv1509 with included hygromycin (200 μg/ml). After 48 h, the recombinants Ms_Vc and Ms Rv1509 were extracted by centrifugation at 3,000 × g for 10 min at 4°C. The cells were washed, and then the cell pellet was dissolved in SDS-PAGE loading dye and heated for 30 min at 90°C. SDS-PAGE was used to separate the lysed fractions, and Western blot analysis was used to detect Rv1509 protein using rabbit anti-Rv1509 polyclonal antisera. After incubation with IgG-HRP, an anti-rabbit IgG monoclonal antibody labeled with horseradish peroxidase, the blots were observed.

### Growth kinetics

The knock-in *M. smegmatis* (mc^2^155) expressing Rv1509 gene (Ms_Rv1509) and *M. smegmatis* (mc^2^155) vector control (Ms_Vc) cells were grown in 7H9 broth supplemented with 10% OADC, 0.5% glycerol and 0.05% Tween 80 at 37°C and 200 rpm. The growth of bacterial cultures was monitored at OD_600_ every 3 h for 120 h to measure the growth kinetics of wild-type *M. smegmatis*, Ms_Vc, and Ms_Rv1509.

### Scanning electron microscopy

For SEM, samples were prepared using the standard protocol (Yang et al., 2014). The exponential phase bacteria were harvested, centrifuged, and washed with cacodylate buffer. After fixation with 2.5% glutaraldehyde for 2–3 h, pellets were washed with cacodylate buffer and then stained with 1% osmium tetroxide, followed by serial dehydration with ethanol. The sample was subjected to sputter gold coating for imaging under scanning electron microscopy (Zeiss).

### Transmission Electron Microscopy

The bacterial samples were prepared using the standard protocol for Transmission Electron Microscopy (Tizro et al., 2019). The mid-log phase bacteria were washed with 1X PBS and fixed in 4% formaldehyde for 15 min, followed by washing with 1X PBS. The fixed bacteria were then embedded in 0.5% agarose gel. The embedded samples were subjected to sectioning and negative staining for microscopy. The prepared blocks were observed under Transmission Electron Microscopy.

### 2D electrophoresis

The 2D electrophoresis was performed using the standard protocol (Akhtar et al., 2020). Ms_Vc and Ms_Rv1509 cultures were harvested, and the culture pellets were dissolved in a lysis buffer and sonicated for 10 min. This was followed by centrifugation at 13,000 rpm for 25 min at 4°C to separate the pellet and the supernatant. The supernatant was subjected to Trichloro Acetic acid (TCA) precipitation in order to remove the salts from the protein sample. The purified protein was dissolved in the re-hydration buffer, and the protein concentration was estimated using the Bradford assay. Equal concentrations of protein samples were loaded onto 5–8 IPG strips. After two h, the proteins were applied to the IPG strips by adding mineral oil and incubated at room temperature overnight. The next day, isoelectric focusing was done according to the standard protocol. After this, the samples were stored at −80^0^C overnight. The SDS gels of stored IPG strips were run, and protein spots were visualized by staining them with Coomassie Brilliant Blue stain. The protein spots on the 2D gel were analyzed using the software PD QUEST.

### RNA isolation

Ms_Vc and Ms_Rv1509 were grown in 7H9 media for 48 h and pelleted by centrifuging at 4,000 RPM for 10 min. The cell pellet was dissolved in 500 μl of Lysis solution (0.05 M Tris and 0.005 M MgCl_2_) followed by bead beating using glass beads. The lysed solution was centrifuged at 4,000 RPM for 10 min to separate the debris and genetic material. The supernatant was collected and mixed with 800 μl of Trizol and incubated at RT (room temperature) for 5 min, followed by the addition of chloroform and incubated again at RT for 3 min. The samples were centrifuged at 13,000 RPM for 15 min at 4°C, and the upper aqueous phase was then collected without disturbing the interphase. The RNA was precipitated using 500 μl of isopropanol and followed by washing with 70% ethanol. The RNA pellet was then air-dried and dissolved in nuclease-free water.

### RNA library preparation

The depletion of ribosomal RNA was performed using a kit with probes to bacterial ribosomal RNA. Furthermore, the ribo-depleted RNA was fragmented, followed by first and second cDNA synthesis, end repair, 3′ adenylation, adapter ligation, and selective enrichment of adapter-ligated DNA fragments through PCR amplification. The QC was assessed using the RNA 6,000 Pico LabChip Kits on the Agilent 2,100 analyzer. The MID study (16S rDNA by Sanger Sequencing) was performed to check the purity of the provided organism and to make sure that the culture was free of contamination. Finally, cluster generation and sequencing on the Illumina platform generated 2X150 bp pair end reads.

### RNA-seq data analysis

The HISAT2 tool was used to map the high-quality reads to the reference *M. smegmatis* genome and build BAM alignments for each sample. Prior to mapping, His-at-build was used to create a reference genome index (HISAT2-specific indexer program). The input reads were fed to the HISAT2 aligner in FASTQ format, along with the reference genome index. StringTie was used, with the BAM files containing read alignments and the reference GTF file as input. StringTie divides the aligned reads into different loci and then divides each locus into as many isoforms as necessary for subsequent analysis. Following this, StringTie uses a flow network method to build and quantify the final transcripts, starting with the most common transcripts. The assembled transcripts were then annotated, and the expression of known genes was quantified using GTF (gene transfer format) annotation files comprising genes. StringTie was used to construct 6,853 known genes using the alignment and *M. smegmatis* genome gene annotation. Gene ontology (http://www.geneontology.org/) and KEGG pathway databases (https://www.genome.jp/kegg/pathway.html) were used to functionally annotate known genes.

The functions of anticipated CDS were classified using gene ontology assignments. The GO mapping also includes an ontology of specified terminology for gene product features, which are divided into three categories: cellular component, molecular function, and biological processes. The output consists of an assembled gene/transcript GTF and an FPKM file. Following this, sample 1 (Ms_VC) on the left was considered the denominator (or control), and sample 2 (Ms_Rv1509) on the right was considered the numerator (or treated). Thereafter, the fold change was calculated as sample 2/sample 1, and these fold change values were transformed to logarithmic base 2 values. The negative value represents downregulated genes, and positive values represent upregulation or no change in expression genes. The annotation of the known genes was done using two databases—Gene Ontology and the KEGG pathway database. For Gene Ontology (GO) annotation, a gene list was created from the reference GTF file, and then this gene list was uploaded to the Uniprot KB webserver (https://www.uniprot.org/help/uniprotkb) in the Uniprot ID/mapping program. The known gene IDs were mapped to gene IDs available in Uniprot KB for *M.smegmatis* bacteria, thereby giving all associated GO ID, terms, and definitions. In the next step, Pathway annotation was done based on reciprocal blast hits of known cDNA sequences of *M.smegmatis* in relation to database sequences in KEGG. To facilitate this step, the cDNA sequences were downloaded from Ensembl and then uploaded to the KAAS server, and the chosen “prokaryotic” gene was set for annotation. As the gene/transcript abundance file was obtained from the StringTie, the FPKM count for each gene in each sample and/or technical replicates were considered for differential gene expression (DGE). An in-house Perl script was used to bring together the FPKM values of the same gene in two samples of the concerned combination (i.e., control vs. treated).

### Macrophage (RAW264.7) infection studies

Ms_Rv1509 and Ms_Vc were electroporated with a green fluorescent protein (GFP) expressing vector (pSC301). RAW 264.7 cells (3 × 105 cells/well) were seeded in 24 well plates, and cells were infected with GFP expressing Ms_Rv1509 or GFP-expressing Ms_Vc at a multiplicity of infection [MOI] 1:10. To begin with, macrophage infection experiments used 1:5, 1:7, and 1:10 MOI for 4 h. To determine the morphology changes of Ms_Rv1509 in the uptake of macrophages and to explore the virulence, we decided to keep the MOI 10. After 4 h of infection, cells were washed three times with 1X PBS to remove extra-cellular bacteria, and fresh growth media was added to the cells along with gentamycin at a final concentration of 50 μg/ml. Live cell imaging at 40X using EVOS FL Auto2.0 was performed to identify the bacteria inside the macrophages. Images were taken at 0, 24, and 48 hpi. For CFU assay, RAW 264.7 cells were lysed using 0.02% SDS, and 100 μl of the sample was plated on 7H10 plates containing Kanamycin (50 ug/ml) and Hygromycin (100 ug/ml) as selection markers. The samples were plated at 0, 24, 48, and 72 hpi, and CFU was estimated.

### Survivability assay using CFU count

The infected RAW 264.7 cells were lysed using 100 μl of 0.04% SDS for 3–5 min. The lysed cells were diluted with 10 ml autoclaved water, and 100 μl of the sample was plated on (Kan+50, Hygromycin 100) antibiotic plates. The plating was done for 0, 24, 48, and 72 hpi, and CFUs were counted after 4 days of incubation at 37^0^C.

### Nitric oxide detection assay

The levels of NO secreted by macrophages were assessed using a Griess reagent kit for nitric oxide detection (Thermo Fisher Scientific). Briefly, 20μl of Griess reagent N-(1-naphthyl) ethylenediamine dihydrochloride and sulfanilic acid, 150 μl of the test sample, and 130 μl of deionized water mixed and incubated at room temperature was measured for 30 min and the absorbance at 548 nm.

### Estimation of cytokines

The secreted cytokines were estimated from the Ms_Vc and Ms_Rv1509 infected macrophages according to the protocol described previously (Arora et al., 2020b). RAW 264.7 cells (3 × 10^5^ cells/well) were infected with GFP-expressing Ms_Rv1509 or GFP-expressing Ms_Vc at multiplicity of infection [MOI] 1:10. After 24, 48, and 72 hpi, supernatants were collected and the levels of IL-12, IL-6, and TNFα were estimated using BD Elisa kits according to the manufacturer's protocol.

### Cell necrosis assay (LDH detection) and Nitric oxide detection assay

The cell necrosis assay was performed to measure the percentage of necrotic cells using the Peirce LDH assay kit (Thermo Fisher Scientific). Briefly, the cell culture supernatants of macrophages infected with Ms_Rv1509 or Ms_Vc were collected at 24, 48, and 72 hpi. Fifty μl of culture supernatant was transferred to a 96-well plate, and 50 μl of LDH assay reagent was added to each well, mixed, and incubated at room temperature for 30 min, followed by the addition of 50 μl stop solution. The absorbance was measured at 490 nm and 680 nm.

### Immunofluorescence staining

Macrophage (RAW264.7) cells were seeded (2 × 10^5^/well) on a coverslip in 24 well plates. The macrophages were then infected with Ms_Rv1509 or Ms_Vc at MOI of 1:10 for 4 h, followed by washing and the addition of complete growth media supplemented with 50 ug/ml Gentamycin. The cells were fixed using 4% formaldehyde at different time points. After fixation, cells were incubated with anti-rabbit LAMP1 and Rab5 antibodies (1:250 dilution in PBS) for 2 h at room temperature in the dark. The cells were then washed with 1X PBS three times, followed by the addition of anti-rabbit IgG (Alexaflour 594, 1:1,000 dilution) along with DAPI for 90 min at room temperature. The cells were then washed and mounted with 90% glycerol and incubated overnight at room temperature, and the next day, images were acquired at 63X and 100X magnification (Oil immersion) using a ZEISS Fluorescence microscope.

### Mice infection studies

The mice study was designed to investigate the survival, immune modulation, and histopathological changes in mice (C57BL/6J). Each group had 5 mice and a total of 3 groups (PBS group excluded) and 2 time points—day 30 and day 90 post-infection. Ms_Rv1509, Ms_Vc, and BCG were grown at 37°C in a shaking incubator in the respective media till the OD reached 0.4–0.6. The cultures were harvested and washed with 1X PBS two times. The calculated bacteria were then injected into C57BL mice at 3 × 10^7^ bacilli/mice (Sweeney et al., 2011) into the intraperitoneum using an insulin syringe. Each group contained 5 mice, along with the uninfected control. Mice were sacrificed on day 30 and day 90 to check the bacterial load in different organs. Spleen and peritoneal macrophages were collected at all the time points, and a CFU assay was carried out to detect the bacterial burden. The liver, pancreas, and lungs were also collected along with spleen and peritoneal macrophages for CFU.

### Flow cytometry analysis

The collected spleen and peritoneal macrophages were stained for FACS analysis using standard protocol. To lyse the red blood cells from the spleen, the RBC Lysis buffer (Thermos Fisher) was used for 5 min, followed by washing with 1X PBS. Then, the cells were blocked with Fc Block solution (BD Biosciences) for 10 min and then washed with PBS. The FACS antibodies were added to the cells (1 × 10^6^) per sample, followed by incubation on ice for 30 min. The stained cells were washed two times with PBS, and FACS readings were taken using BD FACS canto II. The data were analyzed using the Flow Jo Software.

### Histological analysis of mice tissues

After 30 and 90 days of infection with Ms_Vc, Ms_Rv1509, and BCG, the infected mice were killed. Organs such as the lung, pancreas, and spleen were preserved in 4% formaldehyde. The organs were divided into 1.5 × 1.0 cm slices with a surgical knife for histological analysis. After that, each specimen was labeled independently and sent to the automated tissue processor. The tissue processor processed the tissue automatically overnight for 12 cycles, which included a 10% formalin change followed by graded dehydration in 70% alcohol, 80% alcohol, and 90% alcohol, respectively. To complete the dehydration, the tissue was transferred to 100% alcohol, then to liquid chloroform for cleaning, followed by molten paraffin for embedding. After that, the attached tissues were processed in the TEC2800 cryo console to create blocks. Using a rotary microtome (Leica Biosystems Inc., USA), the blocks were sectioned into 4 μm sections. The sections were placed on glass slides for staining and rehydrated using 90% alcohol, 80% alcohol, and 70% alcohol, respectively, before being immersed in distilled water. For examination, the sections were stained with hematoxylin and eosin.

### Ethical statement

All experiments involving lab animals were carried out in accordance with the Committee for the Purpose of Control and Supervision on Experiments on Animals (CPCSEA) guidelines (www.envfor.nic.in/divisions/awd/cpcsealaboratory.pdf) and protocols approved by the Institutional Biosafety Committee and Institutional Animal Ethics Committee of the National Institute of Pathology (NIP), New Delhi, India (Approval No. NIP/IAEC-1505). All animal studies were carried out in positive-pressure units at the National Institute of Pathology under ambient circumstances (25°C, 12 h light/dark cycle).

### Statistical analysis

The data were reported as mean ±SD, and the analysis was done using the student's *t-*test whenever possible. The significance level was set at *p* < 0.05.

## Data availability statement

The datasets presented in this study can be found in online repositories. The names of the repository/repositories and accession number(s) can be found below: NCBI - GSE126837.

## Ethics statement

The animal study was approved by the Control and Supervision on Experiments on Animals (CPCSEA) guidelines (www.envfor.nic.in/divisions/awd/cpcsealaboratory.pdf) and protocols approved by the Institutional Biosafety Committee and Institutional Animal Ethics Committee of the National Institute of Pathology (NIP), New Delhi, India (Approval No. NIP/IAEC-1505). The study was conducted in accordance with the local legislation and institutional requirements.

## Author contributions

PM: Conceptualization, Data curation, Formal analysis, Investigation, Methodology, Validation, Writing – original draft, Writing – review & editing. JA: Conceptualization, Data curation, Formal analysis, Investigation, Methodology, Writing – review & editing. JS: Data curation, Formal analysis, Investigation, Methodology, Writing – review & editing. AR: Data curation, Formal analysis, Investigation, Methodology, Software, Writing – review & editing. JS: Data curation, Formal analysis, Investigation, Software, Writing – review & editing. SZ: Data curation, Formal analysis, Investigation, Validation, Writing – review & editing. YA: Data curation, Formal analysis, Investigation, Writing – review & editing. AA: Data curation, Formal analysis, Writing – review & editing. SH: Conceptualization, Formal analysis, Funding acquisition, Project administration, Resources, Supervision, Visualization, Writing – review & editing. NE: Conceptualization, Funding acquisition, Project administration, Resources, Supervision, Visualization, Writing – review & editing.

## Supplementary material

Supplementary Figure S1 Purified recombinant Rv1509 protein has methyltransferase activity. **(A)** The Rv1509 protein exhibits the S-adenosylmethionine binding site, methyltransferase motif, and DNA-binding leucine zipper motif as predicted by *in-silico* tools. **(B)** *M.tb* protein Rv1509 was purified and detected by Western blot using anti-his antibody. **(C)** *In-vitro* methyltransferase activity assay of purified Rv1509 protein with varying concentrations of the proteins. **(D)** Fluorescence emission spectra showing non-specific binding of Rv1509 protein to DNA. **(E)** Time-based DNA binding assay of Rv1509 protein. **(E)** Deep-learning based AlphaFold structural prediction model of Rv1509 protein.

Supplementary Figure S2 SEM analysis of Ms_Vc and Ms_Rv1509 to determine the length of bacteria. SEM image depicting 95% of the bacilli showing variation in length of the bacteria.

Supplementary Figure S3 Differential expression of genes involved in a cellular component, molecular function, and biological process in Ms_Rv1509 as compared to Ms_Vc using RNA-seq.

Supplementary Figure S4 Cell fusion and swelling of the nucleus of macrophages infected with Ms_Rv1509. RAW 264.7 cells infected with Ms_Rv1509 showed cell swelling as well as cell nucleus enlargement compared to cells infected with Ms_Vc (vector control). GFP (bacteria), DAPI (nucleus of macrophages), and LAMP1(lysosomal marker).

Supplementary Figure S5 Histopathology of Pancreas, Lung, and Liver of mice at 90-day post-infection. **(A)** Represents the tissue morphology of Pancreas, Lung, and Spleen of C57BL/6J mice infected with Ms_Vc, Ms_Rv1509, and BCG after 30-day post-infection (HandE stained, Hematoxylin- Nucleus- Blue, Eosin- Cytoplasm- pink). **(B)** This figure illustrates the spleen size in the dissected C57 mice after infection with Ms_Vc, Ms_Rv1509, and BCG at 90 days post-infection.

Supplementary Figure S6 Increased CD3 population in mice infected with Ms_Rv1509. **(A)** FACS plots explaining the presence of CD3 cells from total T cells at 30-day and 90-day post-infection of C57 mice with Ms_Vc, Ms_Rv1509, and BCG. Graphs **(B, C)** show the percentage and number of CD3 cells at 30 and 90 days post-infection (*p* ≤ 0.05 =^*^, *p* ≤ 0.01 =^**^, and *p* ≤ 0.001= ^***^).

Supplementary Figure S7 High CD8 population in C57 mice due to infection with Ms_Rv1509. **(A)** The FACS plots explaining the activation of CD4 and CD8 at 30 days and 90 days post-infection of C57 mice with Ms_Vc, Ms_Rv1509, and BCG. The bar graphs **(B, C)** show the percentage of CD4 and CD8 cells at 30 and 90 days post-infection (*p* ≤ 0.05 =^*^, *p* ≤ 0.01 =^**^, and *p* ≤ 0.001= ^***^).

Supplementary Figure S8 CD25 cell population in CD4 subsets of mice infected with Ms_Vc, Ms_Rv1509, and BCG. **(A)** The FACS plots explain the activation of CD4+CD25 at 30 days and 90 days post-infection of C57 mice with Ms_Vc, Ms_Rv1509, and BCG. **(B, C)** The graphs explain the activation of CD4+CD25 at 30 days and 90 days post-infection of C57 mice with Ms_Vc, Ms_Rv1509, and BCG.

Supplementary Figure S9 Ms_Rv1509 induces expression of CD25 on T cells and modulates secretion of Th1 cytokines. **(A, B)** A bar graph showing the percentage of CD4+ and CD8+ T cells expressing CD25 marker as measured by flow cytometry. **(C)** Levels of IL-12 measured 30 and 90 days post-infection. **(D–F)** Levels of IL-6 secretion, IL-1β secretion, and TNF-α secretion from serum samples of C57BL/6 mice infected with Ms_Vc or Ms_Rv1509 or BCG, 30- and 90-day post-infection (*p* ≤ 0.05 =^*^, *p* ≤ 0.01 =^**^, and *p* ≤ 0.001= ^***^).

Supplementary Figure S10 Molecular cloning, Expression of Rv1509 in *M. smegmatis*, and morphology of Ms_Rv1509. **(A)** Colony PCR showing the positive clones of the pST-Ki vector harboring the Rv1509 gene. **(B)** Confirmation of positive pST-Ki-1509 gene constructs by restriction digestion. **(C)** Confirmation of positive knock-in *M. smegmatis* colonies harboring pST-Ki_1509 by colony PCR. **(D)** Confirmation of expression of Rv1509 in knock-in *M. smegmatis*.
