# Supplementary figures and images for: Expression of a unique M. tuberculosis DNA MTase Rv1509 in M. smegmatis alters the gene expression pattern and enhances virulence
Source: Front Microbiol. 2024 May 13;15:1344857. doi: 10.3389/fmicb.2024.1344857 (PMC11129820; doi:10.3389/fmicb.2024.1344857)

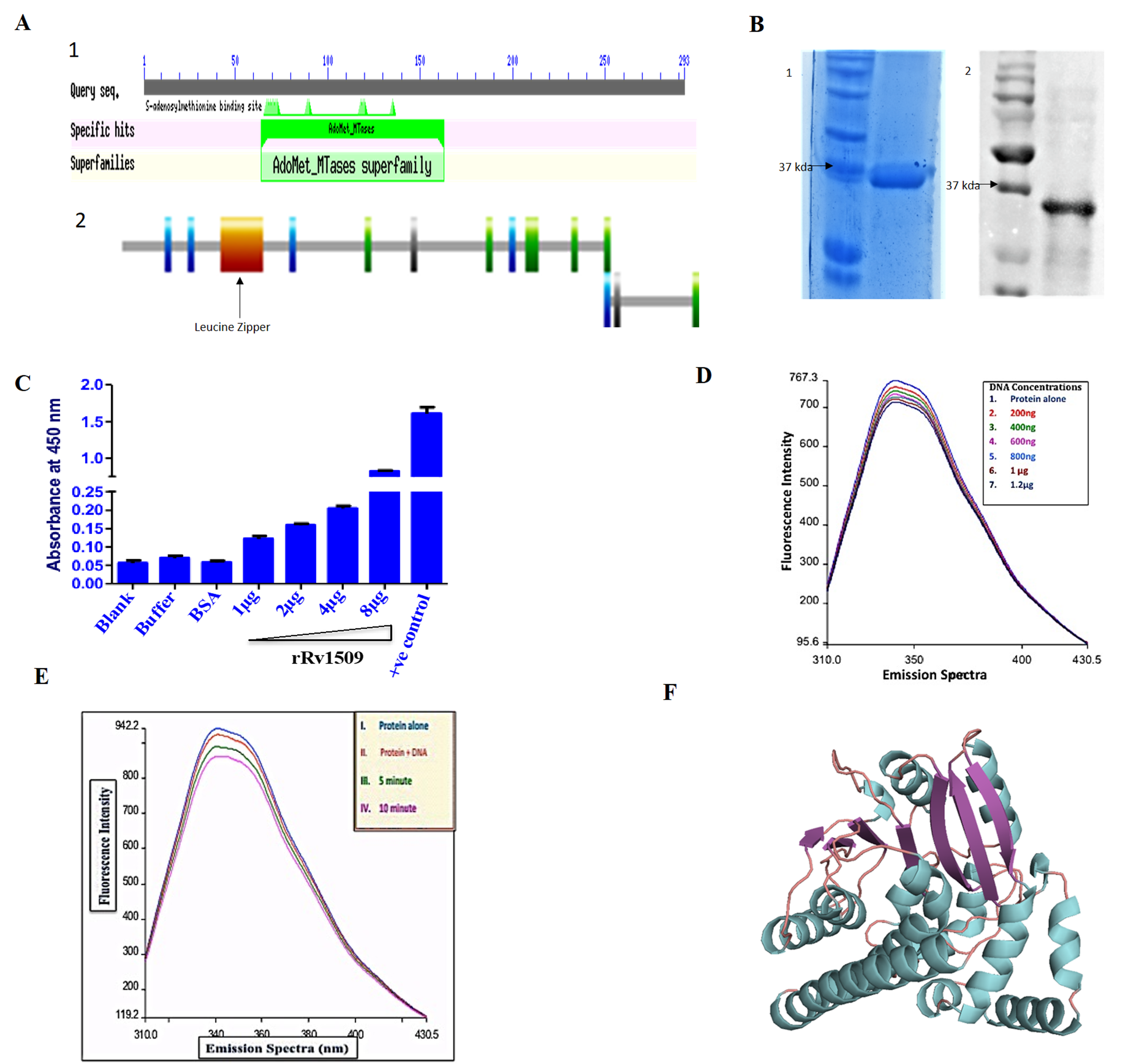

Supplement: Supplementary Figure S1 — Purified recombinant Rv1509 protein has methyltransferase activity. (A) The Rv1509 protein exhibits the S-adenosylmethionine binding site, methyltransferase motif, and DNA-binding leucine zipper motif as predicted by in-silico tools. (B) M.tb protein Rv1509 was purified and detected by Western blot using anti-his antibody. (C) In-vitro methyltransferase activity assay of purified Rv1509 protein with varying concentrations of the proteins. (D) Fluorescence emission spectra showing non-specific binding of Rv1509 protein to DNA. (E) Time-based DNA binding assay of Rv1509 protein. (E) Deep-learning based AlphaFold structural prediction model of Rv1509 protein. [file Image_1.TIF]

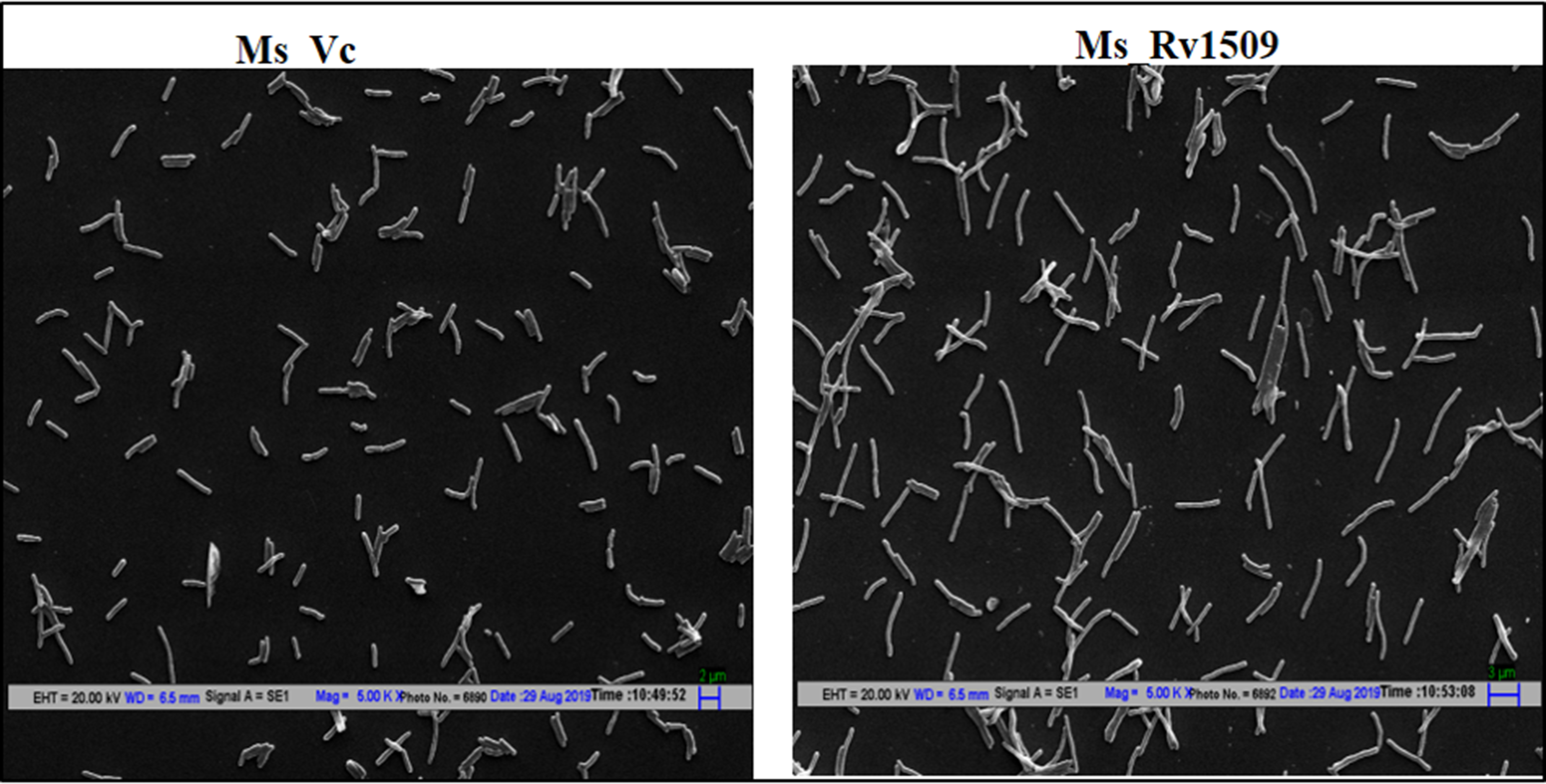

Supplement: Supplementary Figure S2 — SEM analysis of Ms_Vc and Ms_Rv1509 to determine the length of bacteria. SEM image depicting 95% of the bacilli showing variation in length of the bacteria. [file Image_2.TIF]

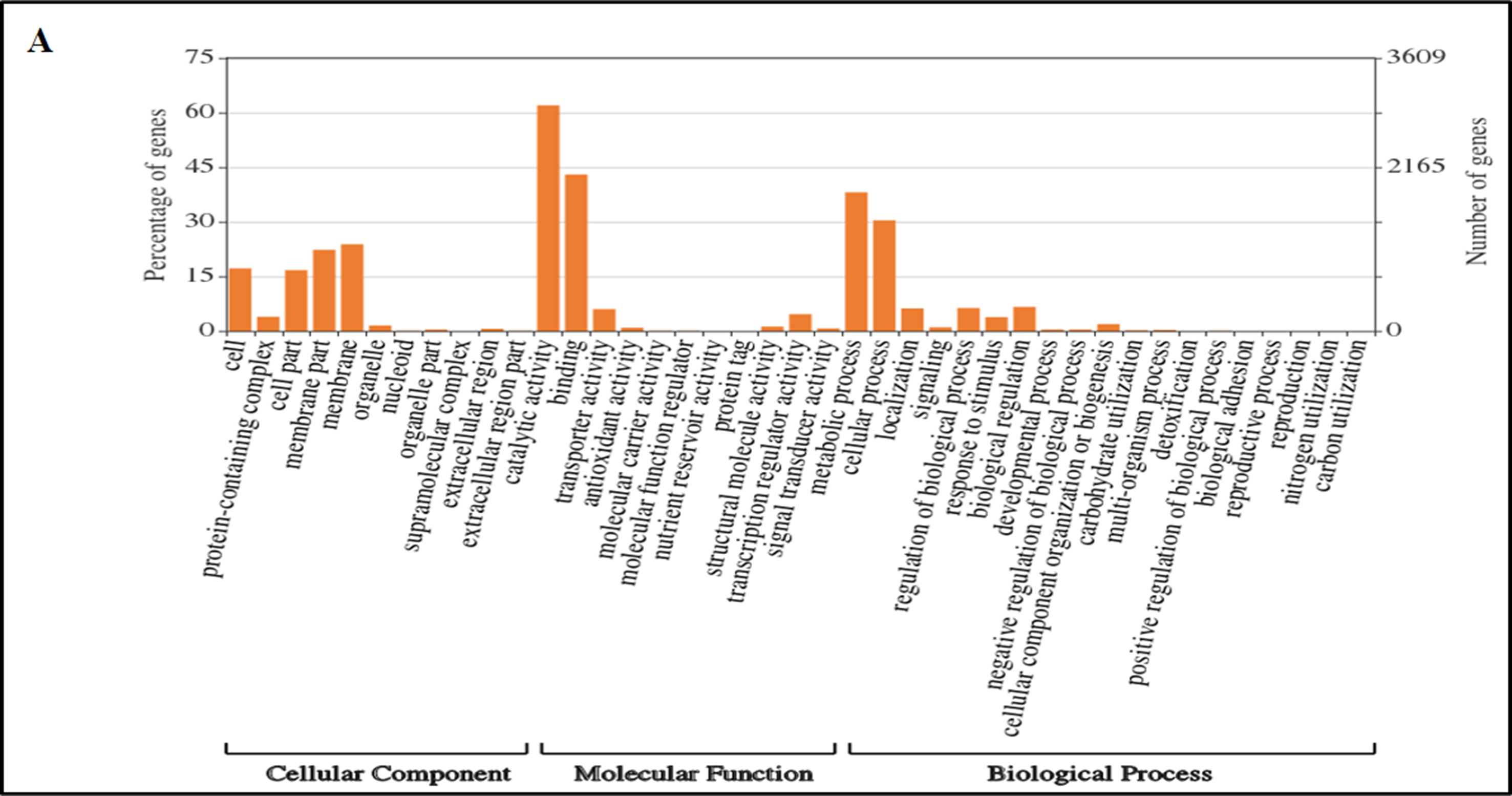

Supplement: Supplementary Figure S3 — Differential expression of genes involved in a cellular component, molecular function, and biological process in Ms_Rv1509 as compared to Ms_Vc using RNA-seq. [file Image_3.TIF]

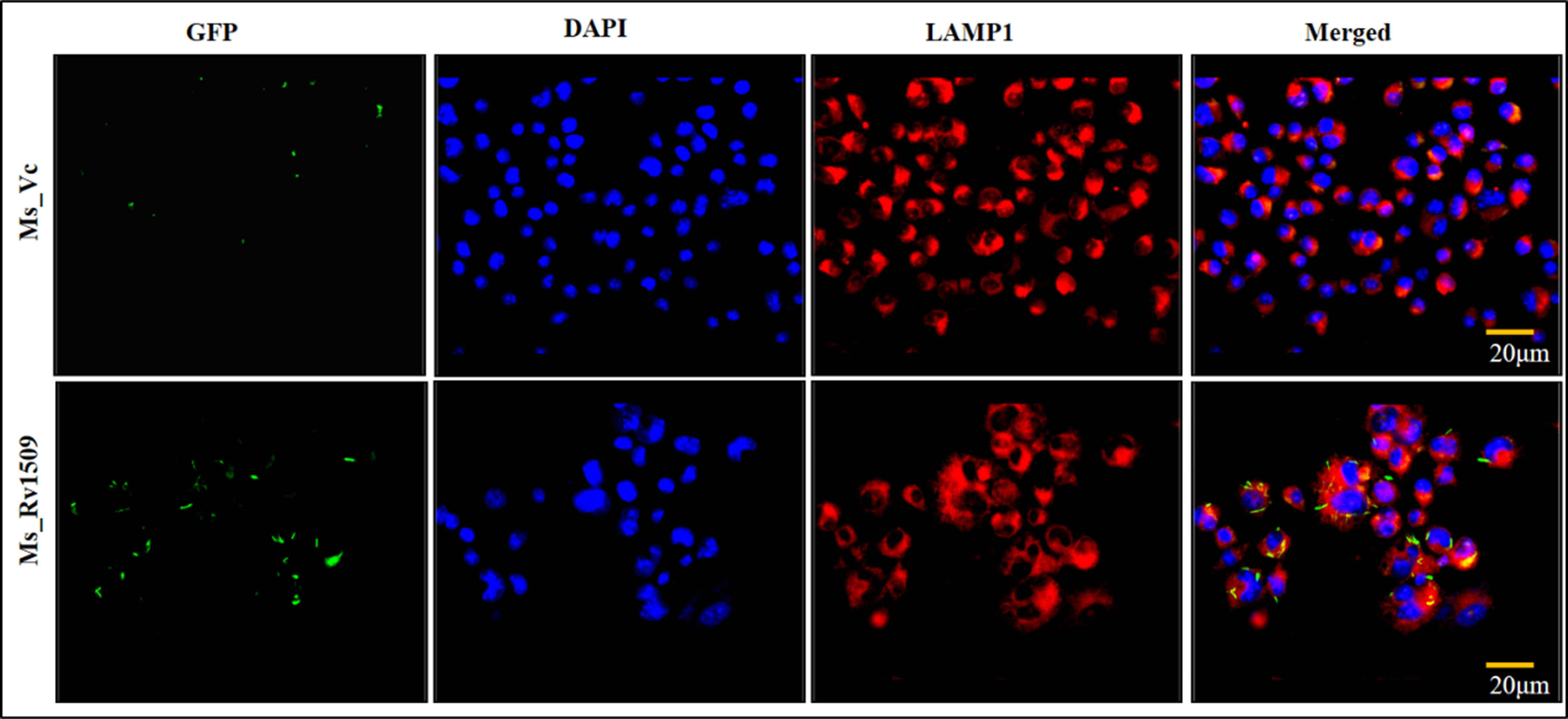

Supplement: Supplementary Figure S4 — Cell fusion and swelling of the nucleus of macrophages infected with Ms_Rv1509. RAW 264.7 cells infected with Ms_Rv1509 showed cell swelling as well as cell nucleus enlargement compared to cells infected with Ms_Vc (vector control). GFP (bacteria), DAPI (nucleus of macrophages), and LAMP1(lysosomal marker). [file Image_4.TIF]

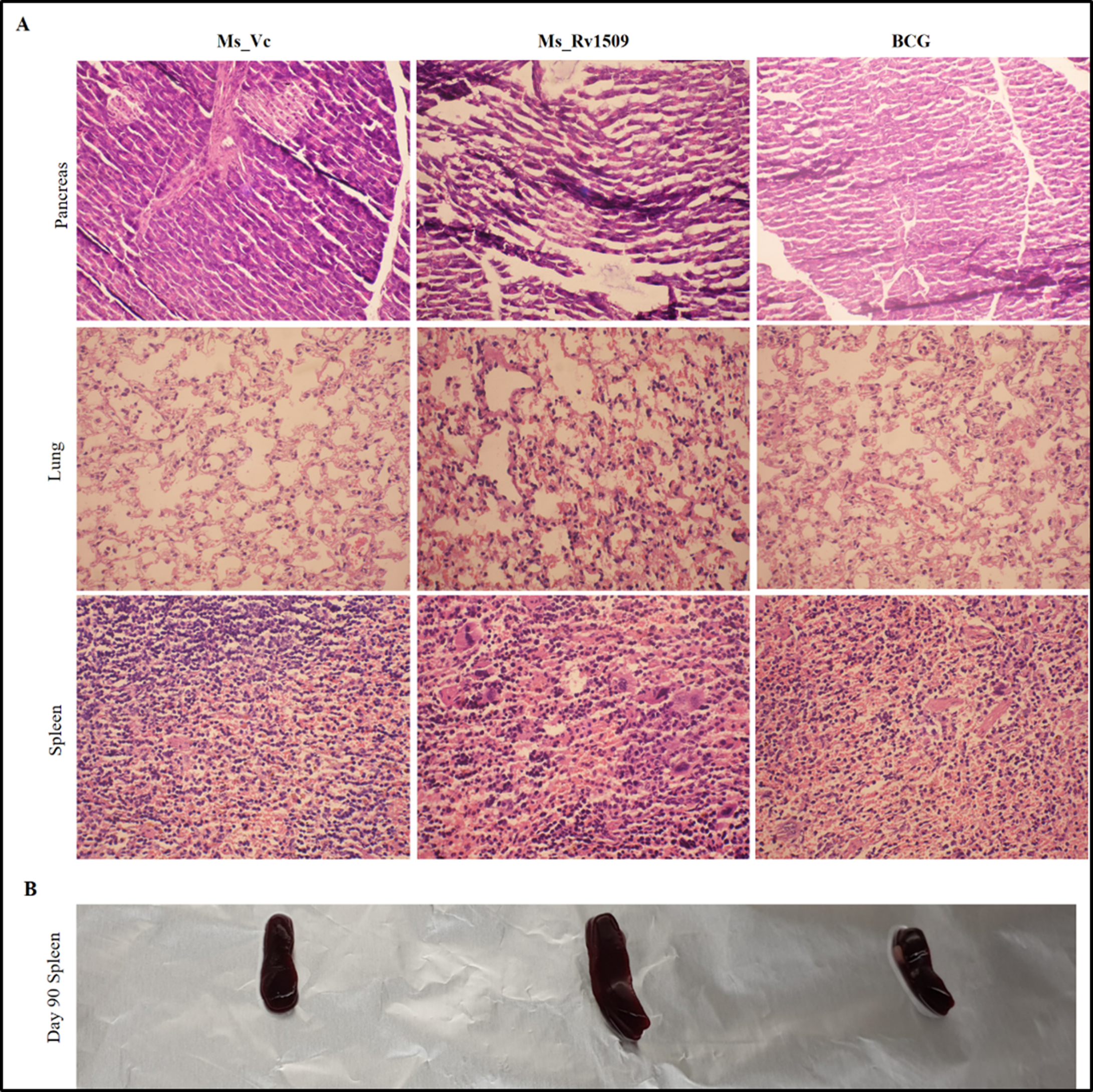

Supplement: Supplementary Figure S5 — Histopathology of Pancreas, Lung, and Liver of mice at 90-day post-infection. (A) Represents the tissue morphology of Pancreas, Lung, and Spleen of C57BL/6J mice infected with Ms_Vc, Ms_Rv1509, and BCG after 30-day post-infection (HandE stained, Hematoxylin- Nucleus- Blue, Eosin- Cytoplasm- pink). (B) This figure illustrates the spleen size in the dissected C57 mice after infection with Ms_Vc, Ms_Rv1509, and BCG at 90 days post-infection. [file Image_5.TIF]

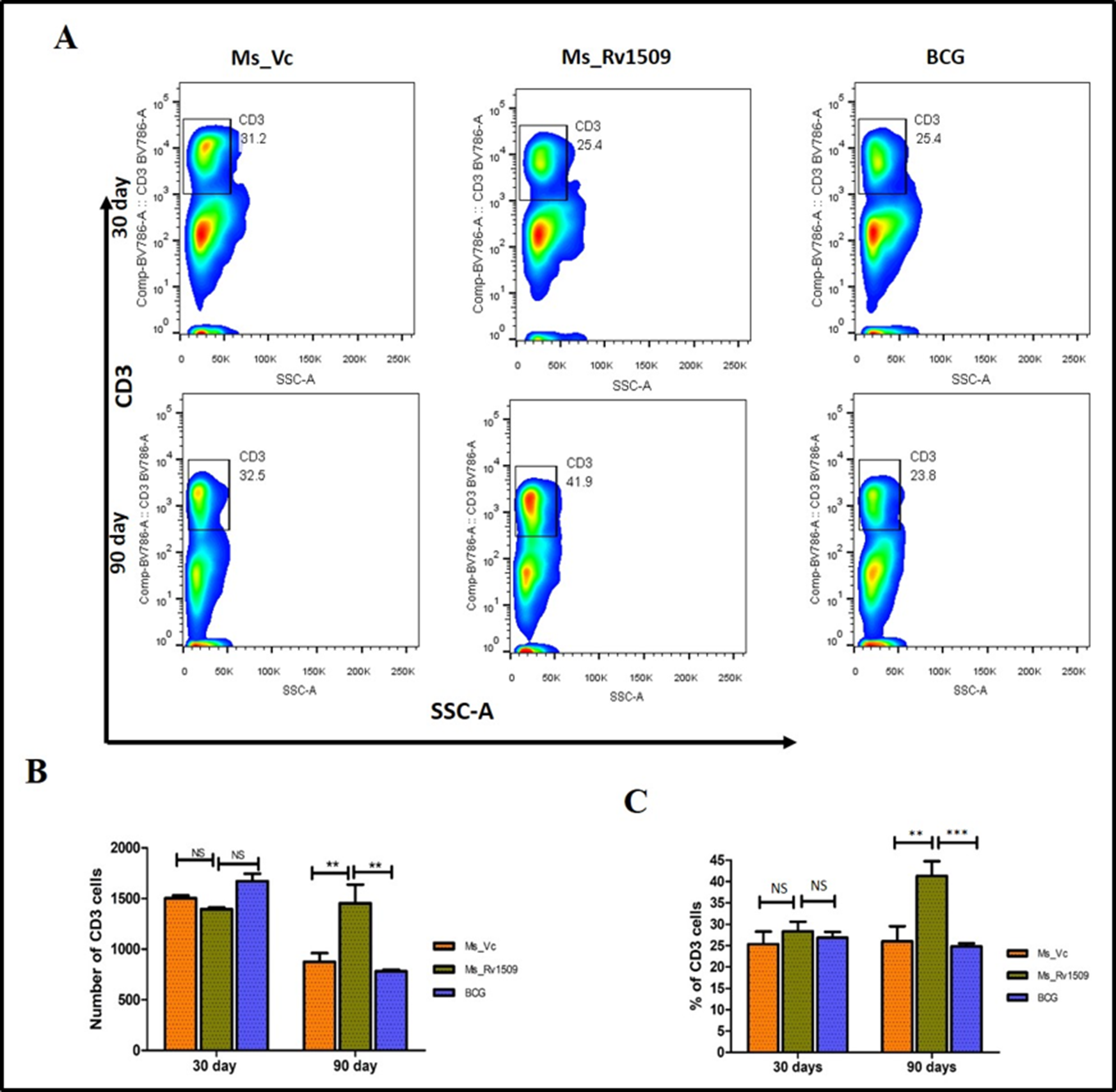

Supplement: Supplementary Figure S6 — Increased CD3 population in mice infected with Ms_Rv1509. (A) FACS plots explaining the presence of CD3 cells from total T cells at 30-day and 90-day post-infection of C57 mice with Ms_Vc, Ms_Rv1509, and BCG. Graphs (B, C) show the percentage and number of CD3 cells at 30 and 90 days post-infection (p ≤ 0.05 =*, p ≤ 0.01 =**, and p ≤ 0.001= ***). [file Image_6.TIF]

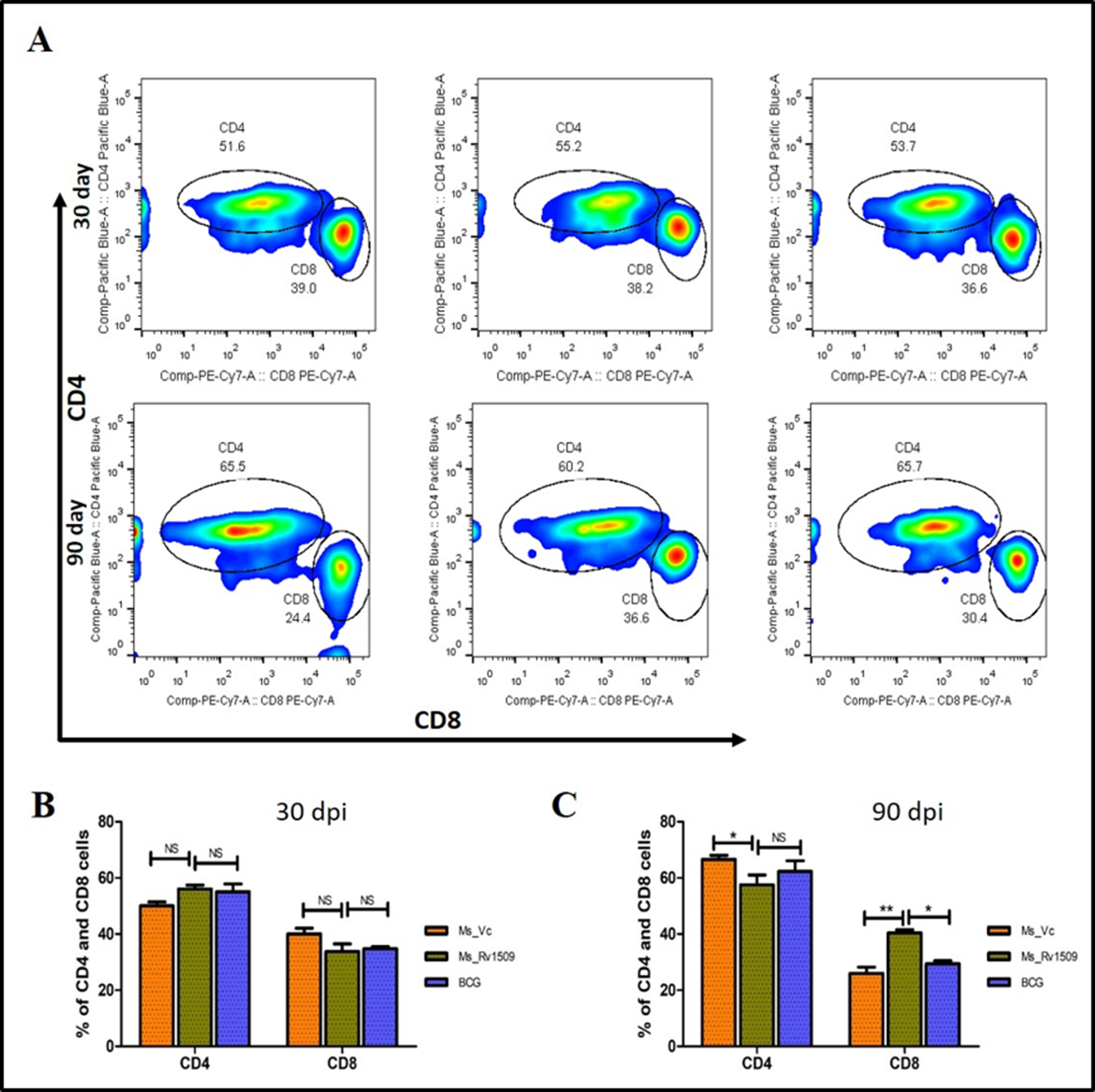

Supplement: Supplementary Figure S7 — High CD8 population in C57 mice due to infection with Ms_Rv1509. (A) The FACS plots explaining the activation of CD4 and CD8 at 30 days and 90 days post-infection of C57 mice with Ms_Vc, Ms_Rv1509, and BCG. The bar graphs (B, C) show the percentage of CD4 and CD8 cells at 30 and 90 days post-infection (p ≤ 0.05 =*, p ≤ 0.01 =**, and p ≤ 0.001= ***). [file Image_7.TIF]

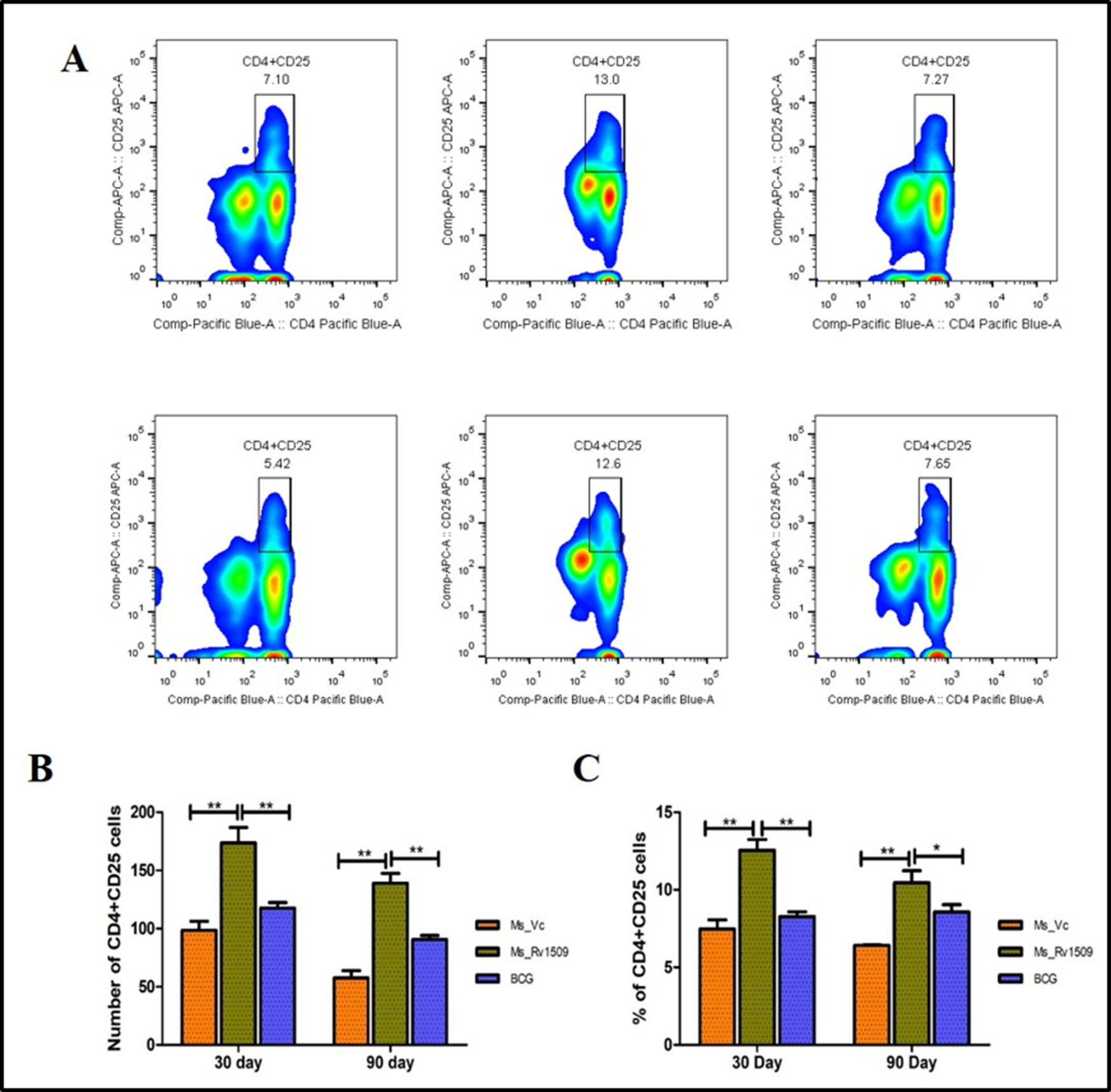

Supplement: Supplementary Figure S8 — CD25 cell population in CD4 subsets of mice infected with Ms_Vc, Ms_Rv1509, and BCG. (A) The FACS plots explain the activation of CD4+CD25 at 30 days and 90 days post-infection of C57 mice with Ms_Vc, Ms_Rv1509, and BCG. (B, C) The graphs explain the activation of CD4+CD25 at 30 days and 90 days post-infection of C57 mice with Ms_Vc, Ms_Rv1509, and BCG. [file Image_8.TIF]

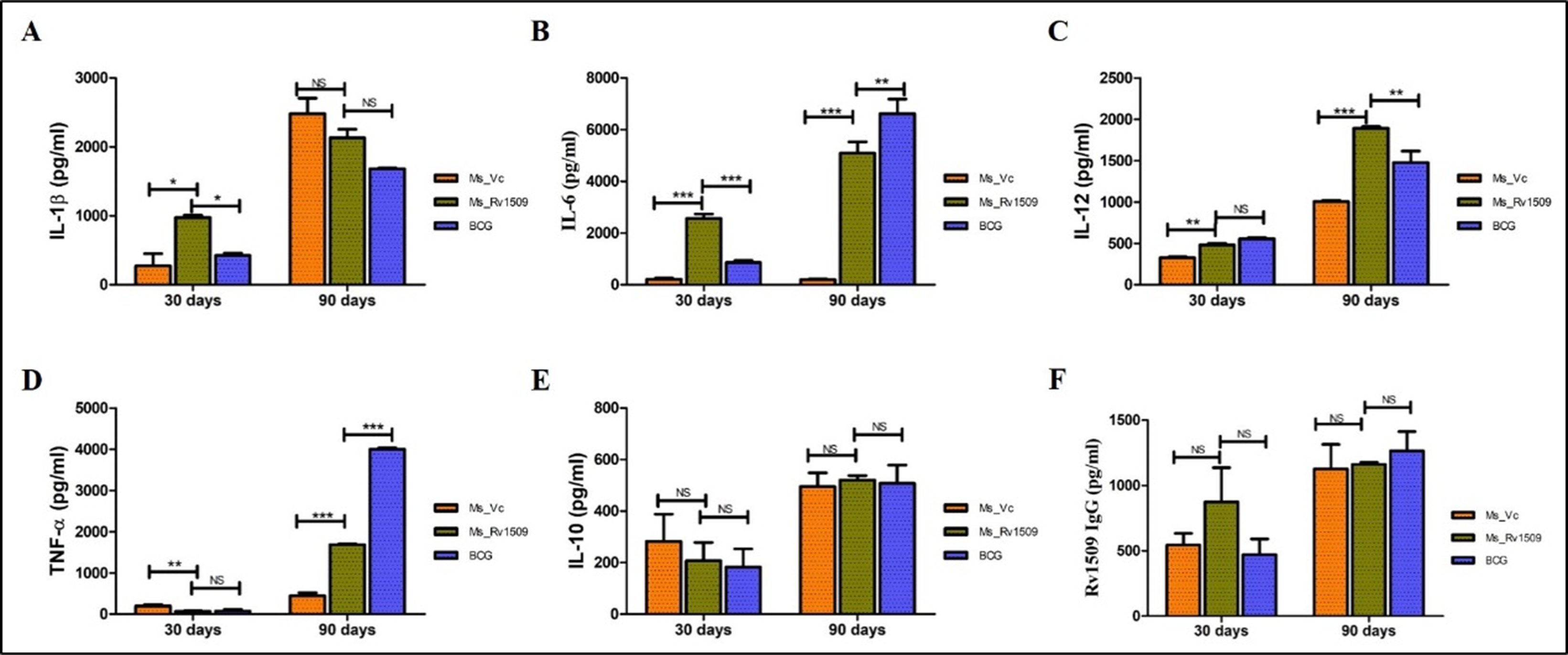

Supplement: Supplementary Figure S9 — Ms_Rv1509 induces expression of CD25 on T cells and modulates secretion of Th1 cytokines. (A, B) A bar graph showing the percentage of CD4+ and CD8+ T cells expressing CD25 marker as measured by flow cytometry. (C) Levels of IL-12 measured 30 and 90 days post-infection. (D–F) Levels of IL-6 secretion, IL-1β secretion, and TNF-α secretion from serum samples of C57BL/6 mice infected with Ms_Vc or Ms_Rv1509 or BCG, 30- and 90-day post-infection (p ≤ 0.05 =*, p ≤ 0.01 =**, and p ≤ 0.001= ***). [file Image_9.TIF]

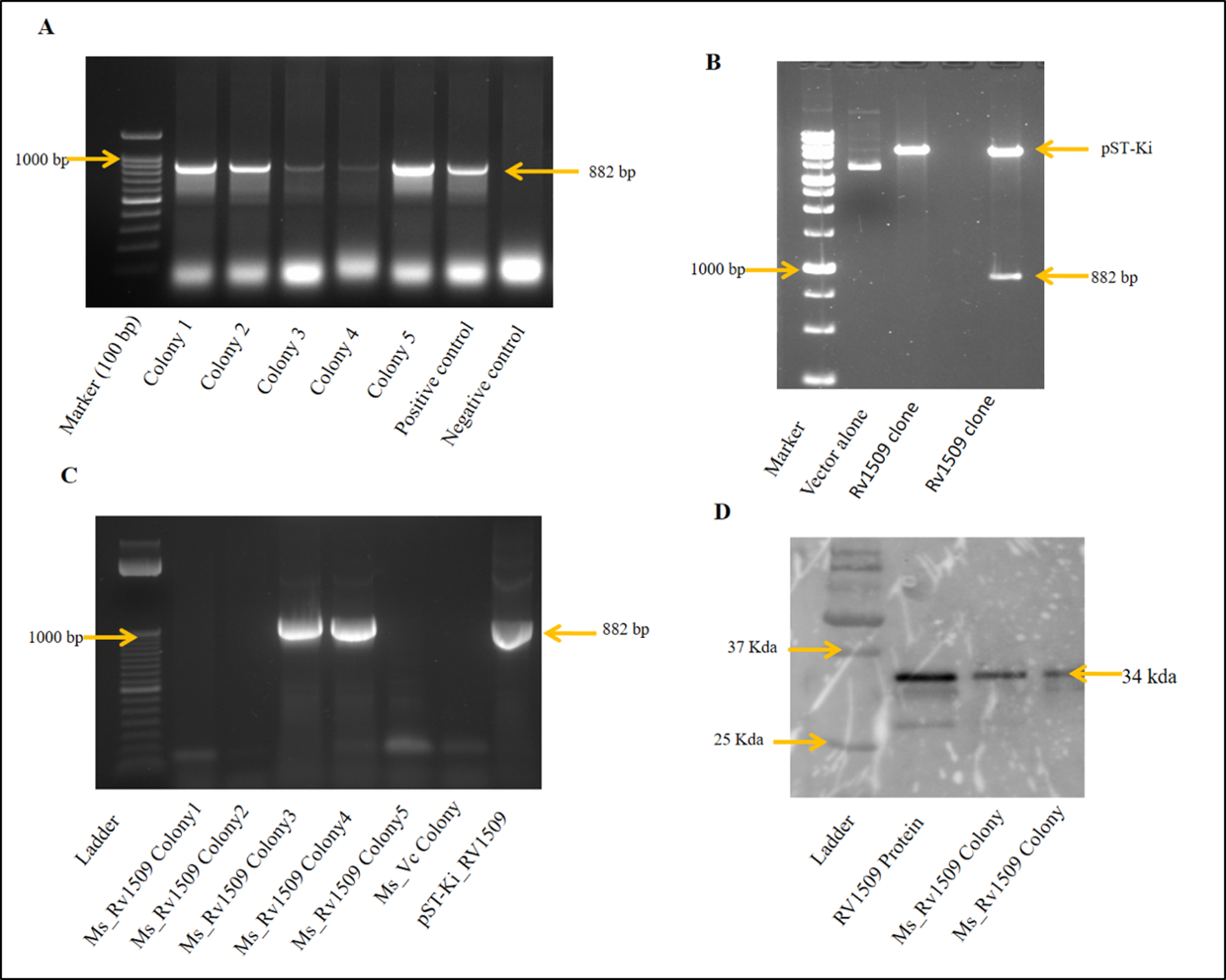

Supplement: Supplementary Figure S10 — Molecular cloning, Expression of Rv1509 in M. smegmatis, and morphology of Ms_Rv1509. (A) Colony PCR showing the positive clones of the pST-Ki vector harboring the Rv1509 gene. (B) Confirmation of positive pST-Ki-1509 gene constructs by restriction digestion. (C) Confirmation of positive knock-in M. smegmatis colonies harboring pST-Ki_1509 by colony PCR. (D) Confirmation of expression of Rv1509 in knock-in M. smegmatis. [file Image_10.TIF]

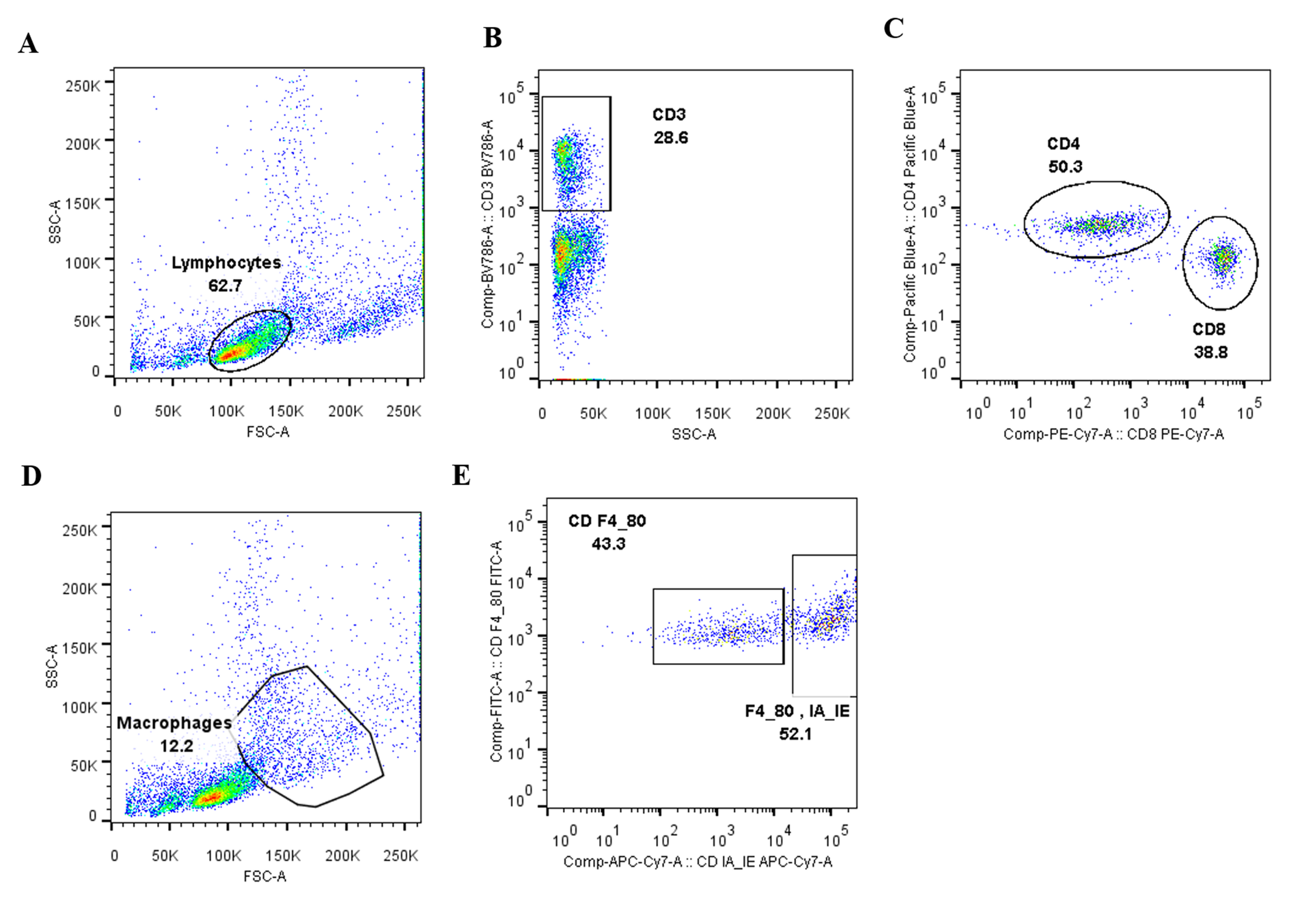

Supplement: Supplementary file 11 [file Image_11.TIF]

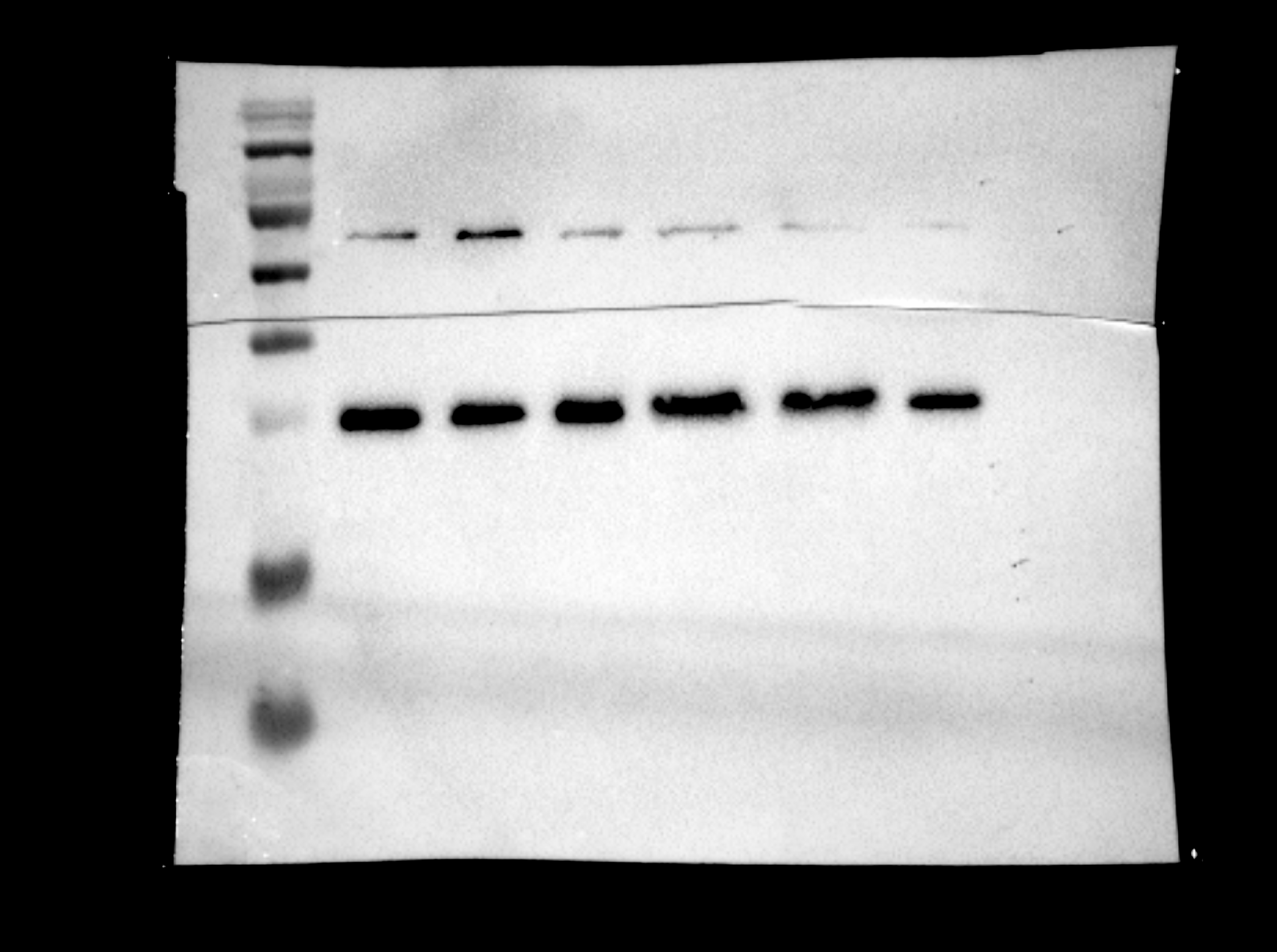

Supplement: Supplementary file 12 [file Image_12.TIF]

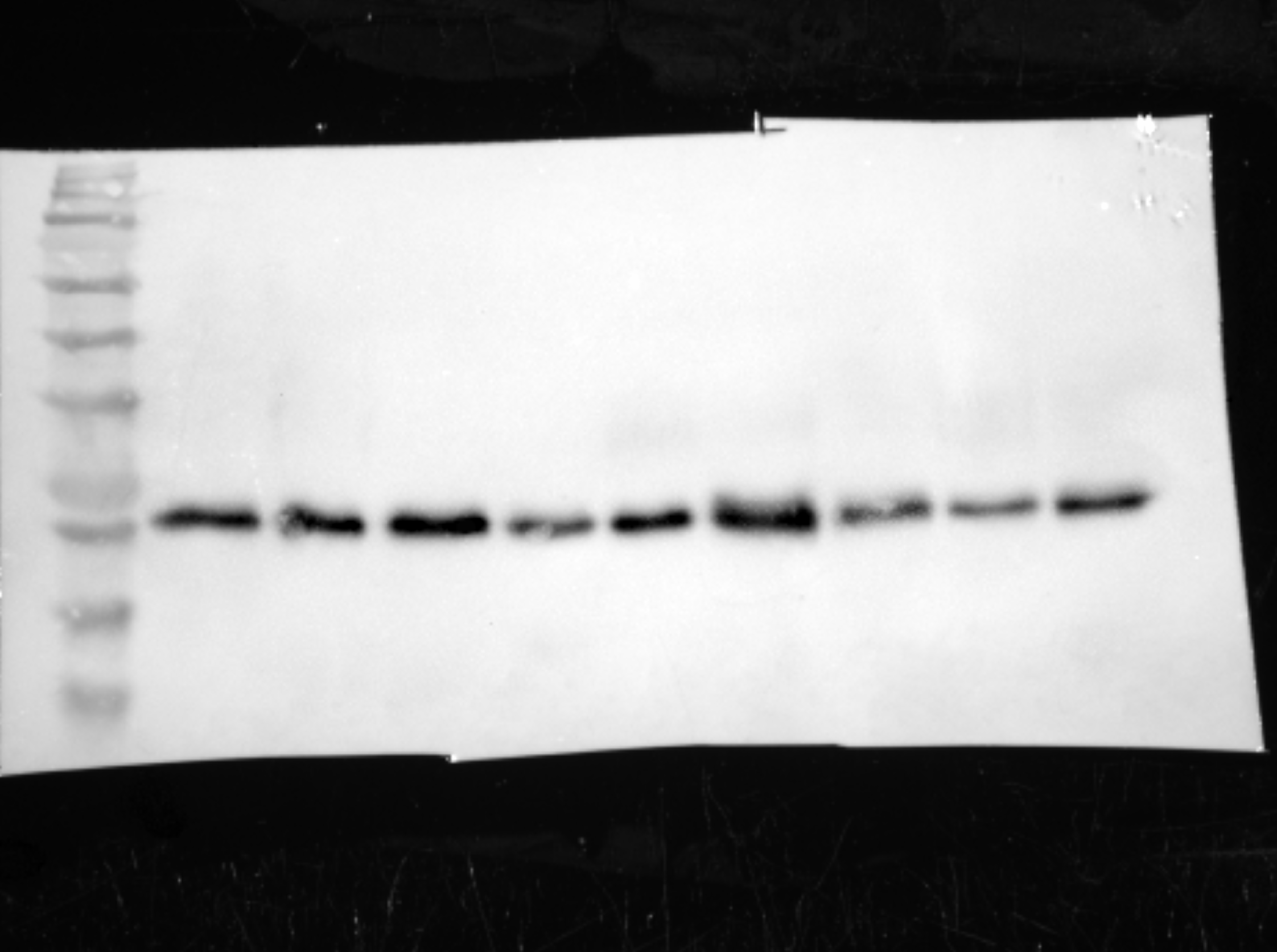

Supplement: Supplementary file 13 [file Image_13.TIF]

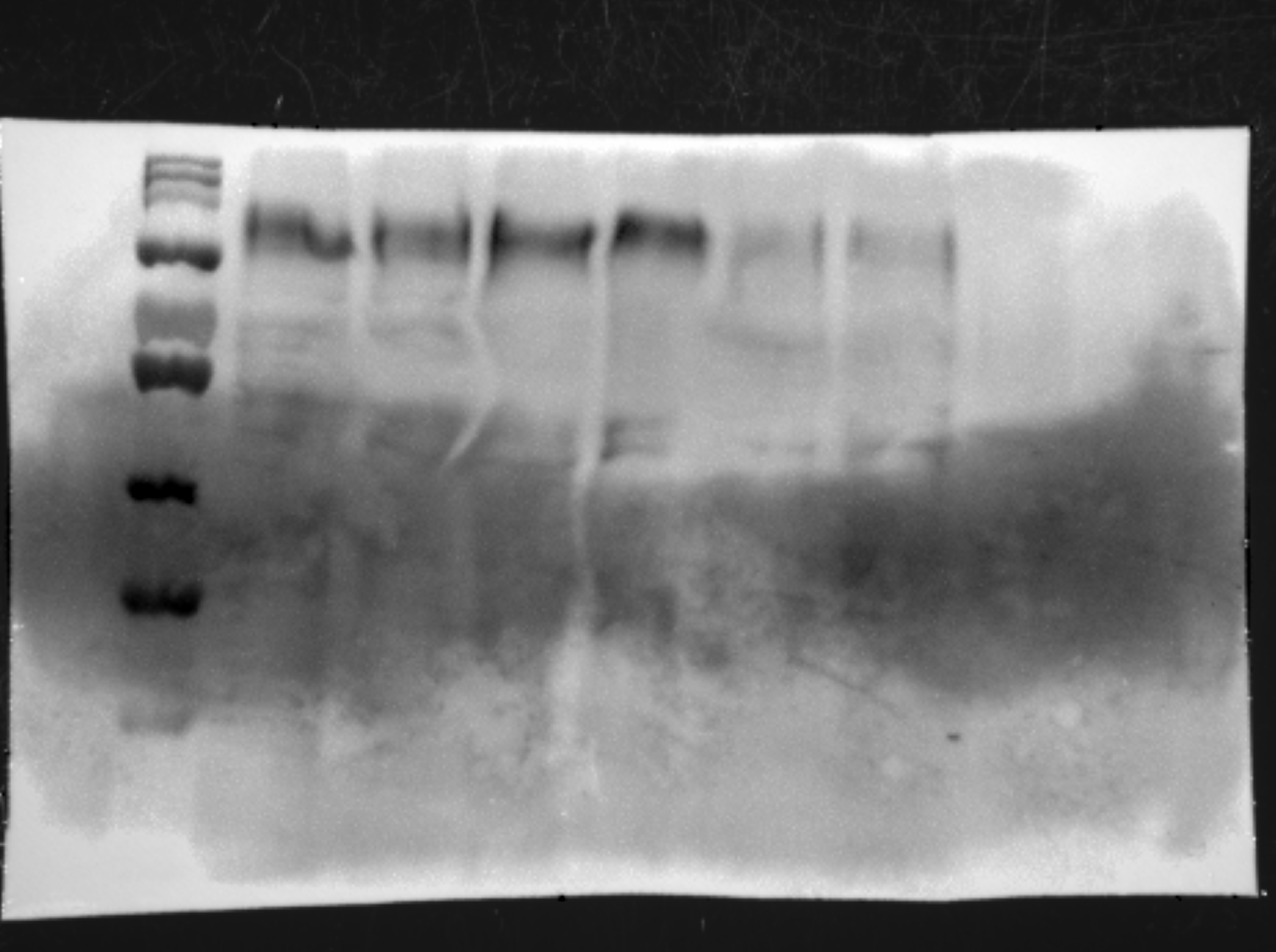

Supplement: Supplementary file 14 [file Image_14.TIF]

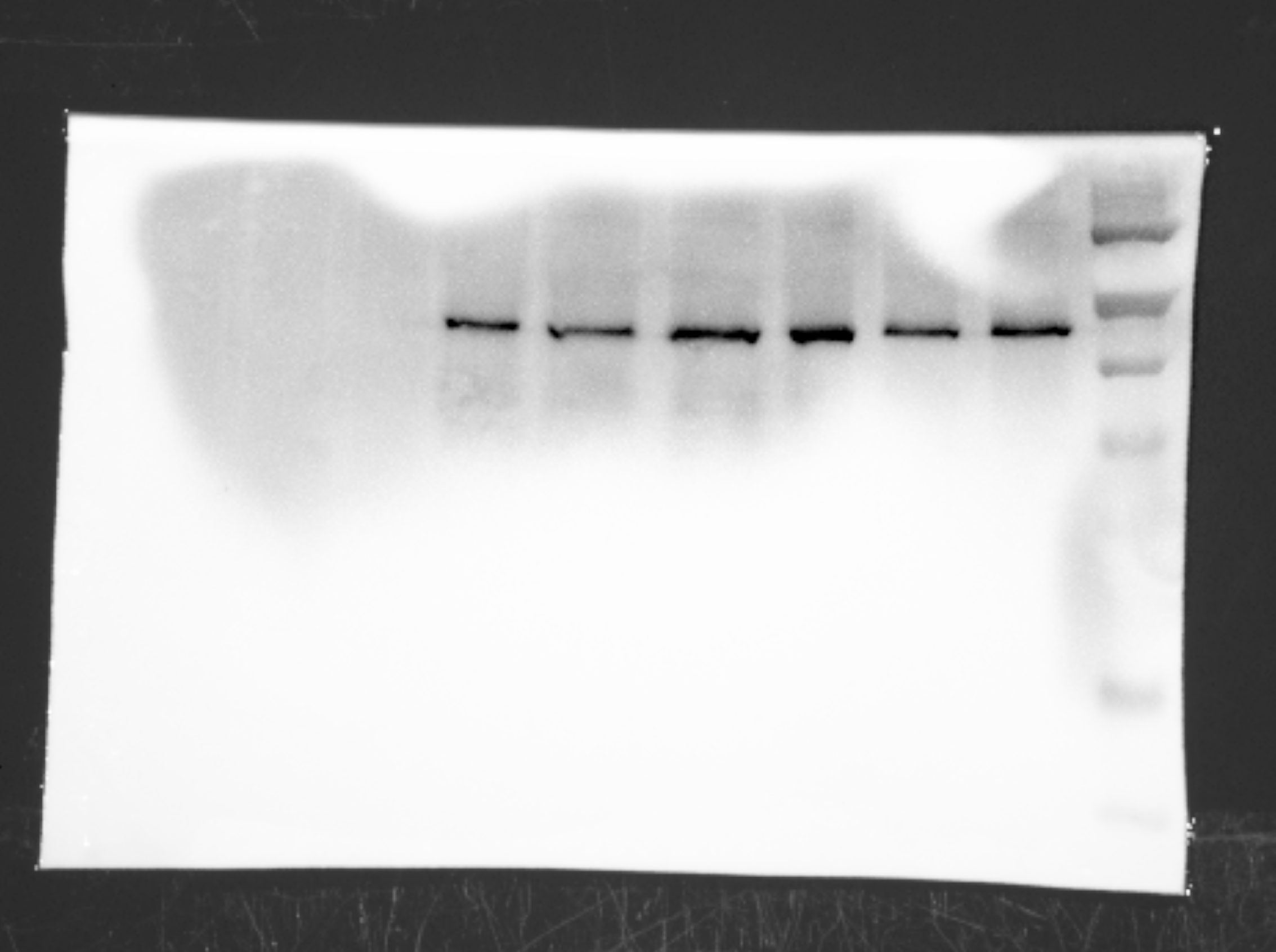

Supplement: Supplementary file 15 [file Image_15.TIF]
