# Supplementary material for: Expression of a unique M. tuberculosis DNA MTase Rv1509 in M. smegmatis alters the gene expression pattern and enhances virulence
Source: Front Microbiol. 2024 May 13;15:1344857. doi: 10.3389/fmicb.2024.1344857 (PMC11129820; doi:10.3389/fmicb.2024.1344857)
Supplement: Supplementary file 18 [file Data_Sheet_1.ZIP › Frontiers FACS data(Raw Files )/Details of samples.docx]

**Day 30 (CD3 and CD25)**- Tube 04- 08 Ms_Vc, Tube 09-13 Ms_Rv1509, Tube 14-18 BCG.

**Day 90 (CD3 and CD25)**- Tube 130- 133 Ms_Vc, Tube 134-137 BCG, Tube 138-141 Ms_Rv1509.

**Day 30 (Macrophages )**- Tube 81-84 Ms_Rv1509, Tube 85-88 Ms_Vc, Tube 89-92 BCG.

**Day 90 (Macrophages )**- Tube 148-149 Ms_Vc, Tube 150-151 MsRv1509, Tube 153-155 BCG.


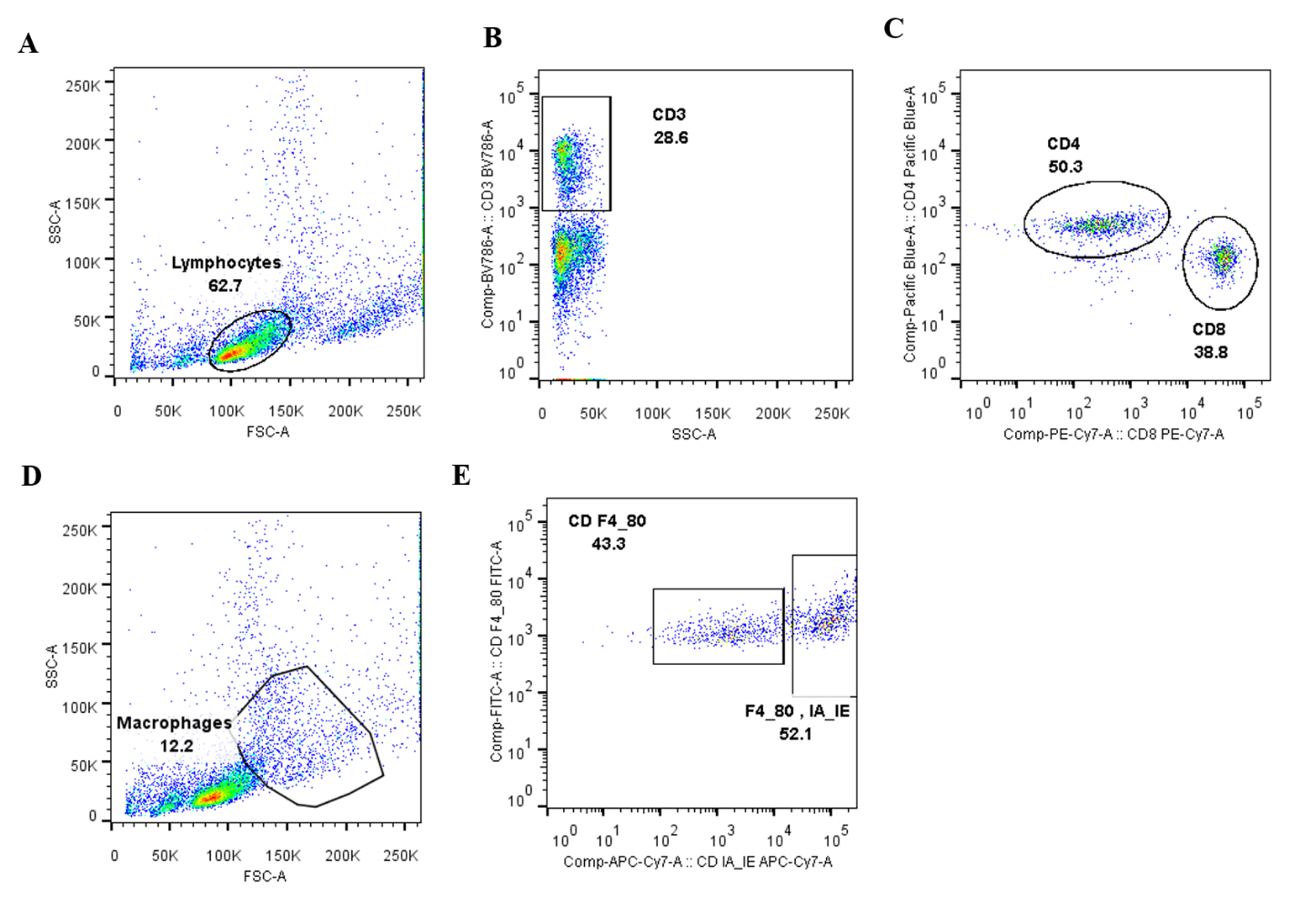


**Figure S11**: **A, B** and **C** FACS plots showing the gating strategy for CD3, CD4 and CD8 markers. **D** and **E** plots explains the gating strategy for macrophage markers.
